# Supplementary material for: “We don’t routinely check vaccination background in adults”: a national qualitative study of barriers and facilitators to vaccine delivery and uptake in adult migrants through UK primary care
Source: BMJ Open. 2022 Oct 10;12(10):e062894. doi: 10.1136/bmjopen-2022-062894 (PMC9557795; doi:10.1136/bmjopen-2022-062894)
Supplement: Author's manuscript [file bmjopen-2022-062894.draft_revisions.pdf]

BMJ Open is committed to open peer review. As part of this commitment we make the peer review history of every article we publish publicly available.

When an article is published we post the peer reviewers' comments and the authors' responses online. We also post the versions of the paper that were used during peer review. These are the versions that the peer review comments apply to.

The versions of the paper that follow are the versions that were submitted during the peer review process. They are not the versions of record or the final published versions. They should not be cited or distributed as the published version of this manuscript.

BMJ Open is an open access journal and the full, final, typeset and author-corrected version of record of the manuscript is available on our site with no access controls, subscription charges or pay-per-view fees (http://bmjopen.bmj.com).

If you have any questions on BMJ Open's open peer review process please email info.bmjopen@bmj.com

BMJ Open

"We don't routinely check vaccination background in adults": A national qualitative study of barriers and facilitators to vaccine delivery and uptake in adult migrants through UK primary care

Journal:	*BMJ Open*
Manuscript ID	bmjopen-2022-062894
Article Type:	Original research
Date Submitted by the Author:	31-Mar-2022

Complete List of Authors:	Carter, Jessica; St George's University of London, Institute for Infection and Immunity<br>Mehrotra, Anushka; St George's University of London, Institute for Infection and Immunity<br>Knights, Felicity; St George's University of London, Institute for Infection and Immunity<br>Deal, Anna; St George's University of London, Institute for Infection and Immunity; LSHTM<br>Crawshaw, Alison; St George's University of London, Institute for Infection and Immunity<br>Farah, Yasmin; St George's University of London, Institute for Infection and Immunity<br>Goldsmith, Lucy; St George's University of London, Infection and Immunity Research Institute, and Population Health Research Institute<br>Wurie, Fatima; UKHSA<br>Ciftci, Yusuf; Doctors of the World UK<br>Majeed, Azeem; Imperial College London, Primary Care<br>Hargreaves, Sally; St George's University of London, Institute for Infection and Immunity

Keywords:	Infection control < INFECTIOUS DISEASES, Public health < INFECTIOUS DISEASES, Health policy < HEALTH SERVICES ADMINISTRATION & MANAGEMENT

SCHOLARONE™ Manuscripts

I, the Submitting Author has the right to grant and does grant on behalf of all authors of the Work (as defined in the below author licence), an exclusive licence and/or a non-exclusive licence for contributions from authors who are: i) UK Crown employees; ii) where BMJ has agreed a CC-BY licence shall apply, and/or iii) in accordance with the terms applicable for US Federal Government officers or employees acting as part of their official duties; on a worldwide, perpetual, irrevocable, royalty-free basis to BMJ Publishing Group Ltd ("BMJ") its licensees and where the relevant Journal is co-owned by BMJ to the co-owners of the Journal, to publish the Work in this journal and any other BMJ products and to exploit all rights, as set out in our licence.

The Submitting Author accepts and understands that any supply made under these terms is made by BMJ to the Submitting Author unless you are acting as an employee on behalf of your employer or a postgraduate student of an affiliated institution which is paying any applicable article publishing charge ("APC") for Open Access articles. Where the Submitting Author wishes to make the Work available on an Open Access basis (and intends to pay the relevant APC), the terms of reuse of such Open Access shall be governed by a Creative Commons licence – details of these licences and which Creative Commons licence will apply to this Work are set out in our licence referred to above.

Other than as permitted in any relevant BMJ Author's Self Archiving Policies, I confirm this Work has not been accepted for publication elsewhere, is not being considered for publication elsewhere and does not duplicate material already published. I confirm all authors consent to publication of this Work and authorise the granting of this licence.

For peer review only

"*We don't routinely check vaccination background in adults*": A national qualitative study of barriers and facilitators to vaccine delivery and uptake in adult migrants through UK primary care

Authors

*Jessica Carter[1] -MBBS jcarter@sgul.ac.uk ORCID 0000-0001-9590-3146

*Anushka Mehrotra[1] MBBS GP anushka.mehrotra@nhs.net

*Felicity Knights[1]- MBBS, MA (Cantab), MPH fknights@sgul.ac.uk ORCID 0000-0002-3007-1438

Anna Deal[1,2] -BSc, MSc, adeal@sgul.ac.uk ORCID: 0000-0001-6168-6542

Alison F Crawshaw[1] – MSc MA Hons. (Oxon), acrawsha@sgul.ac.uk, Orcid ID: 0000-0003-0450-7258

Yasmin Farah BSc[1]

Lucy P Goldsmith – PhD[1]

Fatima Wurie[3]

Yusuf Ciftci[4] PhD

Azeem Majeed[5] a.majeed@imperial.ac.uk, Orcid ID 0000-0002-2357-9858

Sally Hargreaves[1] - PhD FRCPE s.hargreaves@sgul.ac.uk Orcid ID: 0000-0003-2974-4348

***Joint first authors**

[1] The Migrant Health Research Group, Institute for Infection and Immunity, St George's, University of London, London, UK

[2]Faculty of Public Health and Policy, London School of Hygiene and Tropical Medicine, London

[3]Office for Health Improvement and Disparities, Department of Health and Social Care; UK Health Security Agency, London, UK (F Wurie)

[4]Doctors of the World, UK (Y Ciftci)

[5]Department of Primary Care and Public Health, Imperial College London, Charing Cross Campus, The Reynolds Building, St Dunstan's Road, London, W6 8RP, UK (A Majeed)

For peer review only - http://bmjopen.bmj.com/site/about/guidelines.xhtml

Correspondence to: Dr Sally Hargreaves, The Migrant Health Research Group, Institute for Infection and Immunity, St George's, University of London, London, UK s.hargreaves@sgul.ac.uk

For peer review only

For peer review only - http://bmjopen.bmj.com/site/about/guidelines.xhtml

Abstract

- **Objectives:** Explore primary care professionals views around barriers/facilitators to catch-up vaccination in adult migrants(foreign-born; over 18-years) with incomplete/uncertain vaccination status and for routine vaccines to inform development of interventions to improve vaccine-uptake and coverage.
- **Design:** Qualitative interview study with purposive sampling and thematic analysis
- **Setting:** UK primary-care
- **Participants:** 64 primary care professionals (PCPs): 48 clinical-staff including GPs, Practice Nurses, healthcare assistants (HCAs); 16 administrative-staff including practice managers and receptionists (mean age 45 years; 84.4% female; a range of ethnicities).
- **Results:** Participants highlighted direct and indirect barriers to catch-up vaccines in adult migrants who may have missed vaccines as children, missed boosters, and not be aligned with the UK's vaccine schedule, from both personal and service-delivery levels, with themes including: lack of training and knowledge of guidance among staff; unclear or incomplete vaccine records; and lack of incentivization (including financial) and dedicated time and care pathways. Adult migrants were reported as being excluded from many vaccination initiatives, most of which focus exclusively on children. Where delivery models existed, they were diverse and fragmented but included a combination of opportunistic and proactive programmes. PCPs noted that migrants expressed to them a range of views around vaccines, from positivity to uncertainty, to refusal, with specific nationality groups reported as more hesitant about specific vaccines, including MMR.
- **Conclusions:** WHO's new Immunization Agenda (IA2030) called for greater focus to be placed on delivering vaccination across the life-course, targeting under-immunised groups for catch-up vaccination at any age, UK primary care services therefore have a key role. Vaccine uptake in adult migrants could be improved through implementing new financial incentives or inclusion of adult migrant vaccination targets in QOF, strengthening care pathways and training, and working directly with local community-groups to improve understanding around the benefits of vaccination at all ages.

Keywords: Vaccination, migrant, health policy, Europe, COVID-19

For peer review only

Word count: 3500

Article summary

Strengths and limitations

(i) A key strength of the study is the number and variety of primary care staff included from across England in diverse settings.

(ii) Interviewees were a self-selecting group, which may have affected the profile of those responding – a common consideration in qualitative research.

(iii) However, a breadth of practices were involved, including those that do not see many migrants, and this diversity and the scale of the study is likely to have added to the breadth of experience and solutions reflected in our findings, as well as enhancing the validity.

(iv) The structure and experience of primary care across Europe and between the devolved nations of the UK may differ so the recruitment only within England may limit the generalisability of the findings, however we note other European and international studies have come to the similar conclusions.

For peer review only

For peer review only

For peer review only - http://bmjopen.bmj.com/site/about/guidelines.xhtml

Introduction

Adult migrants in Europe – particularly those from low- and middle-income countries – may be at risk of under-immunisation for routine vaccinations due to missed vaccines and doses as children (due to lack of availability, war/disruption, poorly functioning health systems, and personal, social, and physical barriers to accessing vaccines), and/or missed boosters, and differing vaccination schedules in their home country (especially for newer vaccines such as HPV), and so may not be aligned with the UK's vaccination schedule on arrival (1, 2). Additional vaccines may be recommended if they return to their home country, or for specific occupations (eg, tetanus and hepatitis B). Some migrant populations are known to be at risk of under-immunisation (2-5) and were involved in recent outbreaks of vaccine-preventable diseases in Europe, including measles (1). However, adolescent and adult migrants, beyond school age, are often not routinely incorporated into vaccination programmes on arrival to most European countries, including the UK (6). The COVID-19 pandemic has highlighted shortfalls in engaging older migrants, and other marginalised groups, in vaccination programmes (7), yet it has also presented a range of new opportunities and innovations in vaccine service delivery and policy making to these groups, which merit greater consideration beyond the pandemic.

The World Health Organization's new Immunization Agenda 2030 (IA2030) (8) aims to improve vaccine coverage for vaccine-preventable diseases, placing an emphasis on achieving equitable access for vulnerable populations and integrating catch-up vaccination for missed vaccines and doses throughout the life-course. WHO recommends that all countries have a catch-up vaccination policy and catch-up vaccination schedule in place, to close immunization gaps that would otherwise compound as populations increase in age (9, 10), and that it is always "better to vaccinate late than never". Although age limits apply for administration of a small number of vaccines, for most VPDs, providing vaccines late will still result in protection against morbidity and mortality, as well as reducing transmission and risk of outbreaks, with personal and community-level benefits. Specific WHO guidance for catch-up vaccination is available (9); in Europe the European Centre for Disease Prevention and Control (ECDC) has published guidance on catch-up vaccination in children and adult migrants on arrival (11, 12), calling for healthcare providers to consider revaccinating adult migrants with uncertain vaccination status or no recorded history of vaccination. For UK arrivals,

For peer review only

advice is available from the UKHSA on the ‘vaccination of individuals with uncertain or incomplete immunisation status’ (see Panel 1), which will be relevant to most arriving migrants (13). However, the extent to which these guidelines and policies are put into practice and prioritised by UK primary care – tasked with delivering the majority of the UK’s vaccine programme – is not known. No studies to date have explored the views and experiences of front-line primary care teams on approaches to catch-up adult vaccination in arriving migrants. We therefore did a national qualitative in-depth interview study with a range of primary care professionals to understand the challenges and needs of migrant populations with regards to catch-up vaccinations programmes, and facilitators and solutions to addressing gaps in service provision.

For peer review only

Panel 1: Vaccination of individuals with uncertain or incomplete immunisation status. Reproduced from (13).

From tenth birthday onwards:

- Td/IPV and MenACWY*and MMR
  Four week gap
  Td/IPV and MMR
  Four week gap
  Td/IPV

First booster of Td/IPV – preferably 5 years following completion of primary course. Second booster of Td/IPV - ideally 10 years (minimum 5 years) following first booster.

- HPV:

-all females who have been eligible remain so up to their 25th birthday

-males born on or after 01/09/2006 are eligible up to their 25th birthday

- Subsequent vaccination – as per UK schedule (see Flu Vaccine, Shingles vaccine, PPV) and COVID-19.

**Those aged from 10 years up to 25 years who have never received a MenC-containing vaccine should be offered MenACWY. Those aged 10 years up to 25 years may be eligible or may shortly become eligible for MenACWY usually given around 14 years of age. Those born on/after 01/09/1996 remain eligible for MenACWY until their 25th birthday.*

For peer review only - http://bmjopen.bmj.com/site/about/guidelines.xhtml

Methods

Design

Qualitative semi-structured interviews of both clinical and administrative staff were undertaken by telephone, following a topic guide collaboratively developed by the research team with support from a board of migrant representatives. The guide was piloted prior to data collection and iteratively developed throughout the data collection process, with the addition of further prompts and probes to develop richer understanding and addressed key areas around approach to vaccination of adult migrants, factors affecting vaccine hesitancy and uptake, and possible interventions to strengthen delivery (Panel 2).

Ethics and informed consent

Ethics was granted by St George's, University of London Research Ethics Committee (2020.00630) and the Health Research Authority (REC 20/HRA/1674). Participant information sheets were circulated, and signed informed consent was acquired prior to telephone interview. Participants consented to audio record interviews.

Patient and public involvement

During the study design the migrant advisory board were involved in discussion of the study aims and helped review study materials providing feedback on documents including interview questions. The findings will be disseminated to study participants via email later in the year.

For peer review only

Panel 2: Topic guide

Background Questions:
- Proportion of migrants seen at practice, migrant health training and experience
- General barriers and facilitators to registration and provision of care for migrant patients

Questions pertaining to Vaccination of Adult Migrants:
- Are you aware of any guidance regarding vaccination and infectious disease screening in migrants?
- Have there been any outbreaks of vaccine preventable diseases or cases of vaccine preventable diseases in your area involving migrants – we are particularly interested in adults? (If yes, what do they think the reasons might be?
- What experience have you had with adult migrant patients and vaccination?

Questions regarding Practice Approach to Vaccination of Adult Migrants:
- How do you approach catch-up vaccination in the adult migrant patient group, specifically ensuring adult migrants are caught up to align with the UK schedule?
- Who is responsible for vaccination at your practice?
- Is there a mechanism at your practice or in your area to engage adult migrants on catch-up vaccination?
- Is there a local catch-up vaccination pathway?
- Do you target any specific groups?

Questions regarding possible interventions to increase uptake of catch-up vaccination in migrants:
- If there are no mechanisms/pathways in place locally do you think there should be?
- What could such a system look like?
- Are you aware of any other interventions relating to vaccination in migrants? If so, what made them successful/ unsuccessful?
- What do you think about a migrant health check, and what vaccinations would be important to cover in this for adult migrants in your view?
- What in your opinion would be the key to a successful intervention/ behaviour change in primary care?

For peer review only

Setting

Participants were recruited from 50 GP practices in urban, suburban, and rural settings across England. Practices were based in one of six local clinical research networks (CRNs) — CRN Kent, Surrey and Sussex; CRN South London; CRN North Thames; CRN North West London; CRN West Midlands; and CRN Greater Manchester with the exception of a practice in Newcastle and another in Oxford.

Participants

Participants were purposively sampled to capture the diversity of experiences in general practice, from administrative and clinical primary care roles, and practices which varied both in size, and urban/rural location, factors which could influence the number of migrant patients and the organisation of care. Recruitment occurred via local Clinical Research Networks, 'word of mouth' invitations from colleagues and a number of primary care newsletters, social media groups and practice manager mailing lists. All participants who expressed an interest in taking part were e-mailed a participant information sheet and consent form and invited to a telephone interview at a time of mutual convenience, with written informed consent being given in advance. £20 vouchers were given as compensation for each participant's time.

Data collection

Telephone interviews, between 30-60 minutes, were carried out by JC (GP) FK, (GP registrar) and AD and AFC (academic researchers). Findings from the initial interviews were discussed across the group and led to the development of additional prompts and lines of questioning in the topic guide, as well as additional lines of questioning for non-clinical participants. All but three of the interviews were digitally recorded and transcribed verbatim. The remaining three were lost through technical error but were typed up from extensive field notes. Transcripts were anonymised with a coded participant number and checked for accuracy. Data collection continued until there was thematic saturation (14) across all core themes as unanimously agreed across the team.

Data Analysis

Data analysis was inductive, based on the stages of thematic analysis (15). The transcripts were read repeatedly by AM (familiarisation) and emerging themes and patterns were identified and discussed with FK and JC who had also previously immersed themselves in the data. Initially, a coding of 10 transcripts on Microsoft Excel by AM allowed identification of emergent themes and discussion with FK and JC. NVivo (version 13) was then used to organise codes and iteratively refine and develop the emerging coding framework through a process of constant comparison, with close attention paid to non-confirmatory cases which contradicted existing themes. The final coding and themes were conceptualised through recurrent discussion by AM, FK, JC and SH. Active reflexivity was attempted from the study's onset, and input from across the multidisciplinary team, with support from the migrant advisory board, facilitated robust discussion throughout.

For peer review only - http://bmjopen.bmj.com/site/about/guidelines.xhtml

Results

In total, 64 interviews were conducted. 48 interviews were held with primary care staff: 25 GPs, 15 practice nurses, seven healthcare assistants (HCAs), one clinical pharmacist, 11 practice managers, and five receptionists. The majority of staff (50 [78.1%]) worked in urban practices. Characteristics of included participants are presented in Table 1.

Table 1: Characteristics of included participants.

Characteristics	Total participants (n=64)
Staff type	General Practitioners (GPs): 25 (39%)<br>Practice Nurses (PNs): 15 (23.5%)<br>Healthcare assistants (HCAs): 7 (11%)<br>Pharmacist: n=1<br>Practice Managers: 11 (17%)<br>Receptionists: 5 (8%)
Ethnicity	African: 4 (6.3%)<br>Other Asian background: 2 (3.1%)<br>Mixed: 3 (4.7%)<br>Other white: 5 (7.8%)<br>Caribbean: 1 (1.6%)<br>Indian: 11 (17.2%)<br>Pakistani: 3 (4.7%)<br>White British: 32 (50%)<br>White Irish: 2 (4.7%)
Age	45 years (SD 11.8 years)
Sex	Female: 54 (84.4%)<br>Male: 10 (15. 6%)

Barriers reported by PCPs to vaccine uptake in adult migrants

Participants reported that their migrant patients express a range of views around vaccines from positivity to uncertainty, to refusal. They highlighted direct and indirect barriers to vaccine uptake at both patient and staff level, as well as specific issues relating to many migrant groups including specific nationalities. Generalised mistrust and misinformation about vaccinations in migrant groups was commonly reported, which was often perceived by PCPs as resistance to information-sharing about the vaccine in question.

For peer review only - http://bmjopen.bmj.com/site/about/guidelines.xhtml

"It's really hard to break through that barrier of… this is the evidence [about this vaccine]… I don't think they're listening… they're thinking… this is someone from my community saying this [other information]. And you're not from my community… I don't know if you have the best interests [in mind]." GP10

Some PCPs gave their views on vaccine acceptance and uptake linked to specific nationalities, and most often reported beliefs or experiences that migrants originating from Eastern Europe, France and Italy, Somalia and Bangladesh tend to be hesitant about vaccines. Fixed negative views around vaccines were most often reported from Eastern European migrants, who were also viewed as having poor vaccination records and as wanting to follow a different vaccination schedule (as per protocols in their home country), with some returning to their own countries to be vaccinated. (Table 2). The doctor-patient relationship was highlighted as a key factor in tackling mistrust and vaccine hesitancy; some PCPs felt it was easier for migrant patients to connect with PCPs from their own communities.

Table 2: Perceptions of staff around acceptance and uptake in specific nationality groups

Participant reporting	Quote
GP	"Yes, I think the MMR would be a good example, where…I can remember a conversation I had with a mum whose friend, who was also Bangladeshi… Their child was off sick and they blamed it on the MMR vaccination, because before the vaccination he was fine…"
GP	...now it's more Bangladeshi, so Somalian was really with the MMR thing. But we still find more Bangladeshi families delaying or refusing the immunisation of their babies……So, yes they always blame… This is too much, the baby is young, we're not sure about the long term effects.
GP	"[The Somalian population]…is a massive concern for us, with regards the patients unfortunately, falsely attributing MMR with an autism link"……." I think it was the belief of autism, but why more in the Somali community than any other minority group, I'm not too sure."
HCA	[The Somalian population are] … very happy to vaccinate as elderly patients. But, [they think]…the children will get something, get over it. And I think with MMR, they do feel that there's side effects. They think that it causes Autism and things like that.
Practice nurse	I don't know where, Somalia or Eritrea that there was only one interpreter in London who could speak their language. Even their care worker obviously could not speak their language. And so, trying to get immunisation history or any history out of these two young men was totally impossible
HCA	I would say that Europeans [migrants], they refuse because they think they've had them, even if it's been a long time and they don't know.
GP	What I have noticed is that when a patient comes from… Eastern European countries… they do come in with a vaccination record. It's usually incomplete… and sometimes we doubt [it is true and], whether…you can pay someone to give you a vaccination record but it actually hasn't happened.

For peer review only - http://bmjopen.bmj.com/site/about/guidelines.xhtml

Indirect barriers, such as mistrust of the NHS system generally and inability to communicate vaccination histories, were felt by participants to reduce the likelihood of migrants accessing catch-up vaccinations. Issues were raised about immigration status and fears about being reported to authorities, and language barriers, including lack of written communication about vaccine services.

"I think immigration status, out of anything, is going to be the main issue. A lot of people that live in this country without status, going to the GP is a massive risk." PN 13

"Language can be a barrier for subtleties of communication, despite language line" GP21
"I think we probably ought to translate that communication [about vaccine programmes] in written Bengali, and perhaps Somali as well." GP10

"There's usually a long wait and possibly a language barrier as well that may stop [people] from communicating or trying to make that appointment" PN 15

Direct health-system barriers to providing catch-up vaccination for adult migrants included lack of training among staff and lack knowledge of guidance around catch-up vaccination. In addition, participants raised the fact that unclear or poorly documented vaccination histories meant staff were unclear as to what to do, as well as highlighting problems with vaccination records not being transferred within the NHS, and a lack of availability of records from migrants' home countries, including limited translation of previous records into English. Some migrants were reported as having different ages recorded, leading to challenges determining vaccine eligibility.

"And we're certainly not being given any records from other countries that might support [vaccination catch-up]… unless the patient is super well-organised and providing that it happens to be in English or a language that's directly transferrable…" Admin 6

"The nurses would need some kind of education in how to complete incomplete vaccination programmes in adults" Admin 12

For peer review only - http://bmjopen.bmj.com/site/about/guidelines.xhtml

"So, no, I'm not aware of any guidance for [vaccination in] migrant people" GP 2

There were also a number of additional barriers to accessing care at staff and system level which were felt to indirectly reduce the likelihood of adult migrant patients being offered and accepting a catch-up vaccine or travel vaccines through the travel clinic. These included a lack of time to carry out proactive catch-up programmes, or to follow up on opportunistic or challenging conversations where a vaccination need was highlighted, especially when using a translator. The financial pressures and impact of vaccination programmes falling outside of current incentive schemes, such as QOF, also impacted on the time available for the programmes.

"It's just time pressure, the way that the general practice is working at the moment unfortunately is reactive…And so, with things like vaccinations, especially if it's catch up or screening, can always wait… [because] you're going to deal with [someone's chest infection or…diabetes] before you deal with their symptomatic screening. GP6

There are "no incentives for catchup vaccination, MMR… especially compared to childhood immunisations and chronic diseases in QOF." (GP16)

Multiple barriers were identified in relation to specific vaccines in the UK schedule, with a summary of key themes, by vaccine, reported in Table 3.

Table 3: Key issues pertaining to adult migrants for specific vaccines in the UK schedule

Disease	Key message	Quote	Professional
**Influenza**	Different roles of staff in process	'Reception staff call…give [clinicians] the list…then the nurses…[and] doctors vaccine them for both child …and adult.'	Admin 13
	Poor engagement across all nationalities	'Non-engagement is across the board… not specifically one population or another'	Admin 14
	Not specific to migrants	'I don't notice a particular difference between migrants and non migrants.'	GP 18
	Not specific to migrants	'[I] can't think of anything specific that we did for migrants, but…we didn't have many of them'	GP 11

For peer review only - http://bmjopen.bmj.com/site/about/guidelines.xhtml

Disease	Key message	Quote	Professional
	Perceived side effects	'[Adults with flu jab] sometimes they don't want it because they said they had it before and they had side effects, so yes, that's the main thing.'	GP 13
	Engagement with specific nationalities	'I think there's a lower rate amongst Turkish population because I think there was a drive...to target that particular group...[The engagement campaign has been] using community leaders.'	GP 2
	Difficulty explaining benefits of flu vaccine when poor understanding of flu	'I find it difficult to convince them that [flu vaccine]… is useful. Because most …[adult migrants] don't understand the concept of flu.'	GP 4
	Older more likely to engage	'We find that, generally, the over 65s will take it and under 65s will have very low uptake…[not sure] it makes a difference with what ethnic background they're from…'	GP 16
	Reasons for refusing include strengthening immune system naturally and history of side effects	'[Adult migrants]… are refusing because they want to have a[immune] system and teach their body to fight against a virus…[or] they had bad side effects'	HCA 4
**Hepatitis A**	Travel or exposure are reasons for vaccination	'We do …[this] when a patient contacts us, because they're either worried about hepatitis or they're thinking they're going to travel [to their home country]'	Admin 13
**Hepatitis B**	Not within schedule	'Vaccination is not within the schedule, so it has to be treated like a private prescription...and [can be] occupation[al] [eg nurses from South India]'	GP 4
	For travel	'We have given it at times … where we felt that was needed because of travel that was going to happen.'	PN 11
**HPV**	Taboo subject for some migrant families	'Doesn't seem to be a very good uptake of it, in the migrant communities that we have…I think an anything remotely to do, within the genitalia area. When you to to discuss that… it's normally a difficult conversation to have with a lot of the migrant families…. I think they find that a bit of a taboo subject… [they] generally come in groups… [with] mum, dad and maybe a couple of children… [which] makes conversations like HPV… more difficult to discuss.'	HCA 6
**Meningococcal**	Catch-up offered during travel clinics	'If [adult migrants have] …not had a meningitis, I will always offer that to them as part of the travel thing'	PN 2
**MMR**	MMR for adult catch-up	'MMR… [is] the only [vaccination]… that's being offered [for adults as catch-up, regardless of migrant status]'	Admin 1

For peer review only - http://bmjopen.bmj.com/site/about/guidelines.xhtml

Disease	Key message	Quote	Professional
	Barriers to vaccinations	'Based on our experiences with the Somali populace who have had an aversion to MMR vaccine….there'll be cultural barriers, language barriers and… some vaccine skepticism in certain groups of the population.'	Admin 15
	False link with autism	'The Somalian population… falsely attributing MMR with an autism link….'	GP 1
	Migrants more likely to accept catch-up MMR	'… because it's incentivised, we've been inviting anyone who hasn't got it recorded on the system… it tends to be a lot of migrants because they don't come with anything…they're quite happy to come in and have it done'	GP 14
	Current catch-up system not fit for purpose, hence opportunistic more likely but rare	'EMIS (an online practice system) is very annoying because every single patient for who it doesn't have MMR date, it says MMR is outstanding…when people have come, especially if they're refugees or asylum seekers, they won't have that paperwork, they won't necessarily have that knowledge … it's very, very difficult to [know what] they haven't had a vaccine….'	GP 2
	Less accepting of catch-up MMR if age > 40 years	'[When migrant patients register]…Especially for under 40, we try to find out, the MMRs, if they have them…If they are young they will accept. But then, the standard for patients over 40, they don't want to anymore.'	HCA 4
**Rubella**	Rubella uptake in pregnancy is good	'Pregnant women….do get rubella… if you say that it will help their child I don't get any opposition to that'	GP 4
**Shingles**	Education is key	'There's not enough education around it.. it's not something as well-known as, say the flu…it's difficult to get [a translator] for every single patient, to educate them what shingles is..so, I think… it's more education that's needed.'	HCA 6
	For older generation	'… for the older [migrant] generation… not many but a few, are quite happy to come and have the singles vaccinations.'	GP 14
**Tetanus**	Lack of vaccine history means if possible exposure they will be given the vaccine anyway	If [migrants]… needed tetanus for some kind of cut off some kind of infection, we'd just give it them regardless… because we don't really know when the last jab was.	GP 14

Fragmented models for vaccine delivery to adult migrants

Almost all clinical staff reported the availability of good catch-up programmes for childhood vaccination among recently arrived migrants, with some PNs specifically quoting the Public Health England Schedule for individuals with uncertain vaccination status. Incentivization for under-5s vaccination included the Quality Outcome Framework (QOF), and well-resourced systems to ensure

children are not missed, including the vaccination record 'red book' and using recall systems to contact patients, such as sending reminder texts. By contrast, adult migrants were often reported as being excluded from vaccination initiatives. One GP stated that over 5's and adults are sometimes assumed to be *"up to date from the country they come from"*, and many staff, especially GPs and administrators, were not aware of any catch-up vaccination programmes for adult migrants.

"We don't routinely check vaccination background in adults" GP 16

"We do catch-up vaccinations for children and young adults who've missed their primary vaccinations, but in terms of adults or people who are arriving to the UK, no" Admin 6

"Ad hoc. We haven't had a particular programme for [adult catch up vaccinations] GP15

Where adult catch-up vaccination was provided, models of delivery were diverse and fragmented, comprising a range of clinics and providers, different staff members (primarily nurses), and a combination of opportunistic and proactive programmes. Providers of catch-up vaccination for adult migrants included: NHS GP practices, detention centres (for undocumented migrants and asylum seekers), migrant-specific or language-specific clinics, private clinics and specialised clinics (e.g. sexual health clinic in China Town), with distinct benefits and challenges.

Detention centres: "Interpreters weren't always readily provided when I was at the detention centres. We found that really difficult and it took several visits [to determine which vaccines were required and these to be given]" PN13

"I can remember a conversation I had with a [Bangladeshi] mum…Their child was off sick and they blamed it on the MMR vaccination…[she] believed her friend over me… they're thinking, … you're not form my community… I don't know if you have… my child's best interests." Whereas the doctor running the Bangladeshi clinic had more trust from their patients "Because she has contact with them regularly…they tend to connect with her very easily, because they feel … this doctor …[makes] the effort to get to know us, by virtue of just doing her clinic every week" GP 10

For peer review only - http://bmjopen.bmj.com/site/about/guidelines.xhtml

Respondents reported vaccinations programmes were a mix of opportunistic and proactive delivery approaches. Proactive programmes included methods such as setting up searches, call and recall systems to contact patients, and targeted campaigns for specific vaccinations (eg flu).

"We run recalls [for adult migrant catch-up] constantly throughout the year. We will target separate cohorts of patients, just so we can make sure we're recalling everybody". Admin 5

Opportunistic usually meant identifying a patient needed a vaccination when they were attending the practice for another reasons. The vaccine could be given immediately, or the patient booked into an appointment at a later date.

"…if I notice and if I remember or have time to mention it, then I encourage people to… [but] they're usually coming with quite a few issues, and we're using an interpreter… there's a lot to cover…[hence no time to cover vaccination]. GP 18

There are also diverse approaches to vaccine delivery between practices, with different staff involved in different aspects of the vaccine programme. However, many programmes are nurse-led, with the practice nurse having main responsibility.

'[It's a] mixture of me, one of the partners, and then the reception staff are the ones who actually call the patients and arrange for them to come in" Admin 13

"If they're struggling to get somebody to agree [to take a specific vaccine]… we get the named GP.. to take responsibility for having that conversation and trying to talk them round" Admin 9

"…our vaccines are really well-run at the practice by one of the nurses in particular. She runs the whole immunisation program, the childrens, the flu, the catch up, everything. So, I would imagine that there's probably a lot going on that I'm not aware of. I suspect and she always goes on updates and is very much aware of new guidance to things so I'm sure that she's probably doing a lot of stuff behind the scenes that I'm not aware of." GP 3

For peer review only

Travel Vaccination and Occupational vaccines

Provision of catch-up vaccines and additional vaccines to adult migrants was also mentioned in the context of travel and occupational requirements. Delivery of travel vaccinations was highlighted by a variety of participants for migrants visiting their home countries and travelling to Haj.

"I think people are very good at knowing they need vaccinations, especially people who have been settled in England for quite a long time and are maybe making an infrequent return visit home to may visit relatives or family elders or to go for a celebration" Admin 6

"They will go for the bare minimum of what is offered, or what they need to have as certificated. If they're doing the pilgrim to the Haj, then they have to get the meningitis. If they… need yellow fever, they'll get the yellow fever…or they just don't have anything." PN 2

Different nationalities were reported as having varying levels of engagement with travel vaccine uptake. One PN reported Bangladeshi families travelling more being 'more engaged' than Middle Eastern people. Another reported Europeans as '*more engaged with travel clinics than...people… from Pakistan, India, Bangladesh and African countries". [PN2]* African patients were described as having a poorer uptake of travel clinics than Europeans "*people returning to DRC or Tanzania…their uptake is poorer than younger European people" PN2.*

Participants noted that travel clinics can also be an opportunity for opportunistic adult catch-up: *"The nurses who do the travel clinics are certainly very switched on to catch-up vaccines and will make sure everybody's up to date with DTP and MenACWY, even if they're not going to a country for which you need ACWY." GP 17*

Travel vaccines were often given privately due to recommendations these should be done outside of the core contract, and this was primarily the case for adults but not children.

"We do … Hepatitis A and then typhoid as part of the core contract. Anything else we direct patients to a private travel clinic" GP 24

For peer review only - http://bmjopen.bmj.com/site/about/guidelines.xhtml

However, there was variability in provision, with one GP stating: *"We don't charge for anything, including malaria pills" (GP 17). This would impact the "migrant population who are going backwards and forwards to their home countries [and] constitute quite a large percentage of patients that we see for travel clinics" (GP 17)*

Occupational vaccines were mentioned as sometimes being provided 'outside the schedule' for healthcare staff, such as nurses.

" [Hepatitis B] vaccination is not within the schedule, so it has to be treated like a private prescription…some of them are nurses …[and they] usually come from the South Indian population. Carers and nurses" GP 4

"We shouldn't be seeing people wanting occupational health-related vaccination, but we do often get people asking for that" PN 1

Strengthening vaccine delivery in UK primary care

Primary care staff raised a range of potential solutions and action points to increasing vaccine uptake, especially in adult migrants, including addressing personal, societal, and physical barriers to vaccination systems through UK primary care alongside financial incentives to primary care to deliver adult catch-up vaccination. Barriers and potential solutions raised by participants are summarised in Table 4.

For peer review only

Table 4: Barriers and solutions identified

Barrier	Potential solution	Key messages	Quotes	Professional
**Awareness of vaccination programmes for adults**	Community engagement, capacity development, investment and partnership-building to raise awareness	Engage with community leaders, faith groups to help GPs and public health systems to improve uptake for vaccines in migrants; provide opportunities for information sharing, outreach, engagement, communication	*'I …hope that the CCG have thought about this and have gone to local communities, through the mosque or through other social avenues to trying get [vaccine] uptake'*	GP 24
**Fear of authorities**	Community engagement to tackle mistrust; increasing trustworthiness of health and other institutions	Education and raising awareness within communities to overcome fear and enable health-seeking of preventative healthcare; (re)building trust through community engagement and investment	*'We have suggested … that they engage with the churches, that they obviously engage with information and advice, but it's a hard nut to crack if somebody's life is built around not trusting the specific institution.'*	GP 24
**Misinformation about vaccines**	Use trusted professionals or other trusted messengers - and ensure they are properly resourced, recognised and compensated(16)	One GP thought that consulting with someone who was felt to be an 'expert' in vaccinations would have better outcomes.	*'…If [the vaccine advice is from] from a GP…[or] from a consultant… then that tends to have a bit more weight to it… I think it depends on the level of education and understanding…'*	GP 25
	System approach - Building capacity to recognize and respond to misinformation; developing resources to increase health literacy;	Public Health messaging and a national approach	*I think it's got to be a national approach…We got the Public Health Department…'*	GP 22
	Patient education ; develop tailored messages	Patient education and sharing as much information as possible regarding vaccines, from all health professionals involved	*'People just need as much information as possible [about the vaccine], and I think information in particular on side effects etc"*	HCA 1

For peer review only - http://bmjopen.bmj.com/site/about/guidelines.xhtml

Barrier	Potential solution	Key messages	Quotes	Professional
**Lack of training for staff around migrant health**	Staff education and training (both clinical and non-clinical staff)	Improving staff understanding of potential issues and communication skills	*It's just a bit of understanding… some patients may come across as difficult… [but with] extra training with staff…[understanding can improve]*	HCA 2
**Financial pressures**	Financial payments and incentives	Including adult migrant vaccination targets as a financial incentive to ensure migrant adult catch-up programmes are carried out	*"…Unless they actually make [adult catch-up vaccination] something that they want GP surgeries to do, like proactively educate them and give them some renumeration to do it.. work is money and we haven't got enough practice nurses as it is…So it can't just be expected to be an add on'*	GP 18
**Lack of time**	Longer appointments	Longer appointments, especially if interpreter is needed	*We make the appointments longer*	PN 7
**Language barrier**	Interpreters; linguistically and culturally tailored information	Use interpreters for vaccine programmes, including written communication	*We sent out a lot of text messages [about vaccination]. That would be good if we could do those in different languages…*	PN 15
**Different vaccine schedules and lack of history**	Migrant specific health check	A health check for adult migrants, to gather information about vaccine history	*'[A] template which is specific for patients from different countries, which means that you're not trawling through evidence'*	GP 20
**Pressures on health system**	Ensure primary care deliver these vaccination programmes	Make migrant adult catch-up vaccination mandatory for primary care to provide	*'If they were part of QOF, they're made mandatory… that would definitely make [practices] do it'*	Pharmacist

For peer review only

Discussion

Key findings

WHO's new Immunization Agenda (IA2030)(8) has called for greater focus to be placed on delivering vaccination across the life-course, targeting under-immunised groups for catch-up vaccination at any age, with primary care services therefore having a key role to play in the UK context. In our study, however, participants highlighted direct and indirect barriers to delivering catch-up vaccines in adult migrants who may have missed vaccines as children, missed boosters, and not be aligned with the UK's vaccine schedule. Barriers were noted at a personal and service-delivery level, with themes including: lack of training and knowledge of guidance around catch-up vaccination among staff; unclear or incomplete vaccine records; and lack of incentivization (including financial reimbursement), prioritisation, and dedicated time and care pathways. Adult migrants were therefore reported as being excluded from many vaccination initiatives, most of which focus exclusively on children. In addition, PCPs reported that migrant patients express a range of views around vaccines to them, from positivity to uncertainty, to refusal. Some migrants including Somali, Eastern-Europeans and Bangladeshi groups were often reported as being hesitant to get vaccinated, with specific concerns reported for specific vaccines, including MMR. Greater consideration needs to be placed on potential delivery points for catch-up vaccination in adult migrants – for example, local places of worship and other trusted or familiar sites – alongside offering financial incentives or inclusion of adult migrant vaccination targets in QOF. Improving uptake of catch-up vaccination in this group will require new care pathways and training of front-line staff, alongside working directly with local community groups to communicate the benefits of vaccination at all ages. In addition, greater collaboration across systems and community groups and culturally competent campaigns are warranted. At a time when COVID-19 vaccination programmes are being rolled-out across the world, this study adds important understanding regarding the specific vaccination needs and concerns of migrants, and the challenges faced by the staff delivering vaccination programmes to migrant populations and older cohorts.

Strengths and limitations

A key strength of the study is the number and variety of primary care staff included from across England in diverse settings. Interviewees were a self-selecting group, which may have affected the profile of those responding – a common consideration in qualitative research. However, a breadth

For peer review only

of practices were involved, including those that do not see many migrants, and this diversity and the scale of the study is likely to have added to the breadth of experience and solutions reflected in our findings, as well as enhancing the validity. We noted that often staff made broad generalisations about specific nationality groups, which needs to be considered when assessing findings. The structure and experience of primary care across Europe and between the devolved nations of the UK may differ so the recruitment only within England may limit the generalisability of the findings, however other European and international studies (6, 17, 18) have come to the similar conclusions in terms of healthcare provider, system, and patient-related barriers to catch-up vaccination in relation to adult migrants, so we feel that this would be unlikely.

Next steps for strengthening catch-up vaccination in older cohorts

We found a range of direct and indirect barriers to delivering catch-up vaccines in adult migrants who may have missed vaccines as children, missed boosters, and not be aligned with the UK's vaccine schedule, from both a personal and service-delivery level. Our findings concur with those of similar study in Norway (17) which found no consistent or structured approach to vaccinating adult migrants in Norway, including no guidelines from governing bodies on how to organise vaccination to adult migrants. Reasons why adult vaccination is not prioritised included tuberculosis screening and treatment taking precedence, and a common assumption among healthcare providers that vaccinations are dealt with in childhood(17). A questionnaire survey of experts across Europe(6), and policy analysis(19), found that policies and practice differ across European countries with respect to adult vaccination and the inclusion of migrants in vaccine systems on arrival. Only 13 of 32 countries in the EU/EEA had policies in place to offer MMR vaccines to adult migrants, with 10 countries reporting that they would charge fees(6). Variations in vaccine policies targeting adult migrants were reported in another European survey (20). In addition, it is well known that some migrants face a range of barriers to health systems more broadly. This suggests that more inclusive policies are required placing an emphasis on new approaches to ensure older migrants are included, and that such policies are well implemented in practice.

Implementation will be key, and our study raised numerous points that merit greater consideration. Service delivery barriers have previously been described in other areas of migrant health, including screening for infection, with GPs citing concerns about lack of awareness around the health needs of migrants and insufficient time and resources (21, 22). It has previously been noted that negative

For peer review only - http://bmjopen.bmj.com/site/about/guidelines.xhtml

biases from healthcare staff towards migrant patients or pre-conceptions about vaccine hesitancy in specific ethnic groups may have an impact on patient trust (23, 24), which is known to be a major factor in vaccine uptake (25). Education and training of front-line providers will be a critical component given the critical role that the PCP-patient relationship has for building trust in vaccination. This must involve raising awareness of the diverse experiences of migrants and how to approach potential vaccination concerns with sensitivity, as well ensuring an understanding around the potential the low vaccine coverage in their countries of origin as children, different dosing schedules, and particularly low coverage for newer vaccines. For HPV, for example, global coverage for the final dose was only 13% in 2021 (26) – suggesting many migrants aged under 25 years would be eligible for HPV vaccination as part of the UK's more advanced programme. However, likely a key factor will be financial incentivisation to encourage practices to target potentially under-immunised adults for catch-up vaccines, which was a recurrent theme among those interviewed. Catch-up vaccination could be considered at various entry points, for example the New Patient Health Check or the NHS Health Check. Since April 2020, MMR now comes with an item of service payment, including for catch-up vaccination in patients who missed out on scheduled vaccines, which should encourage practices to offer appropriate vaccinations to patients regardless of age.

Tackling hesitancy and educating migrant and broader ethnic minority communities about the benefits of vaccination across the life-course will also be a critical component(22, 27), with COVID-19 presenting numerous innovations in service delivery in this area that merit further consideration to routine vaccination going forward including outreach, policy shifts to facilitate registration of migrants with primary care providers, and anonymous vaccination in trusted locations (22, 28). We found that certain nationality groups (Somali, Eastern-Europeans and Bangladeshi) may be more hesitant to receive vaccines than others, or reluctant to receive certain vaccines, aligning with a recent systematic review that found nationality/country of origin to be a key determinant of vaccine uptake for routine vaccines and COVID-19 vaccines in European datasets (7). In this study, acceptance barriers were mostly reported in Eastern European and Muslim migrants for HPV, measles, and influenza vaccines, with 23 significant determinants of under-vaccination in migrants found ($p<0.05$), including African origin, recent migration, and being a refugee/asylum seeker (7). A systematic review of interventions to improve vaccination uptake in newly-arrived migrants to the EU/EEA (29) highlighted the potential solutions of social mobilization and outreach programmes, planned vaccinations, and educational campaigns. Our data points to a

For peer review only - http://bmjopen.bmj.com/site/about/guidelines.xhtml

recommendation for policy makers to include adult migrants specially in catch-up vaccination programmes on arrival, and to ensure policy around the delivery of catch-up vaccination across the life-course is implemented in practice.

Contributors

SH and JC conceived the idea and developed the initial proposal with input from all co-authors. JC wrote the ethics applications, led the data collection, and contributed to data analysis and manuscript revision. AM led the data analysis and contributed to the manuscript draft, revision, and concepts. FK contributed to data collection, data analysis, manuscript draft and revision. AD and AC contributed to the data collection and manuscript revision. All authors contributed to manuscript development and approved the final manuscript.

Competing interest statement

All authors report having nothing to declare.

Funding statement

This work has been funded by the NIHR (NIHR300072). JC is funded by an NIHR-in practice clinical fellowship (NIHR300290). SH, AFC and LPG are funded by the NIHR (NIHR300072), and SH and AFC are additionally funded by the Academy of Medical Sciences (SBF005\1111). SH acknowledges funding from the Novo Nordisk Foundation/La Caixa Foundation (Mobility– Global Medicine and Health Research grant) and the World Health Organization. AD is funded by the MRC (MR/N013638/1). AM is supported by the NIHR Applied Research Collaboration NW London. FK is supported by a Health Education England/NIHR Academic Clinical Fellowship. We acknowledge the support of the European Society of Clinical Microbiology and Infectious Diseases (ESCMID) Study Group for Infections in Travellers and Migrants (ESGITM). FW is a member of the Vulnerable Migrants Wellbeing Project Advisory Board, led by the University of Birmingham and Doctors of the World and funded by the Nuffield Foundation. The funders did not have any direct role in the writing or decision to submit this manuscript for publication. The views expressed are those of the author(s) and not necessarily those of the NHS, the NIHR, or the Department of Health and Social

For peer review only

Care. The funder of the study had no role in study design, data collection, data analysis, data interpretation, or writing of the report.

Data availability statement

All data relevant to the study are included in the article or uploaded as supplementary information.

Acknowledgements

We thank members of our NIHR Patient and Public Involvement Project Advisory Board (Babatunde Tikare, Larysa Agbaso, Monika Hartmann, Saliha Majeed) and additional members of our Project Board for valuable contributions.

References

1. Deal A, Halliday R, Crawshaw AF, Hayward SE, Burnard A, Rustage K, et al. Migration and outbreaks of vaccine-preventable disease in Europe: a systematic review. The Lancet Infectious Diseases. 2021.

2. Mipatrini D, Stefanelli P, Severoni S, Rezza G. Vaccinations in migrants and refugees: a challenge for European health systems. A systematic review of current scientific evidence. Pathogens and Global Health. 2017;111(2):59-68.

3. Jablonka A, Happle C, Grote U, Schleenvoigt BT, Hampel A, Dopfer C, et al. Measles, mumps, rubella, and varicella seroprevalence in refugees in Germany in 2015. Infection. 2016;44(6):781-7.

4. Norman FF, Comeche B, Martínez-Lacalzada M, Pérez-Molina J-A, Gullón B, Monge-Maillo B, et al. Seroprevalence of vaccine-preventable and non-vaccine-preventable infections in migrants in Spain. Journal of Travel Medicine. 2021;28(4).

5. Charania NA, Gaze N, Kung JY, Brooks S. Vaccine-preventable diseases and immunisation coverage among migrants and non-migrants worldwide: A scoping review of published literature, 2006 to 2016. Vaccine. 2019;37(20):2661-9.

6. Hargreaves S, Nellums LB, Ravensbergen SJ, Friedland JS, Stienstra Y, On Behalf Of The ESGITM Working Group On Vaccination In Migrants. Divergent approaches in the vaccination of recently arrived migrants to Europe: a survey of national experts from 32 countries, 2017. Eurosurveillance. 2018;23(41).

7. Crawshaw AF, et al. Defining the determinants of vaccine uptake and under-vaccination in migrant populations in Europe to improve routine and COVID-19 vaccine uptake: a systematic review. 2022 (Accepted, Lancet Infectious Diseases). https://www.medrxiv.org/content/10.1101/2021.11.08.21266058v1.

For peer review only - http://bmjopen.bmj.com/site/about/guidelines.xhtml

8. World Health Organization. Immunization Agenda 2030 2020 [updated 1 April 2020. Available from: https://www.who.int/teams/immunization-vaccines-and-biologicals/strategies/ia2030.
9. WHO. Leave no one behind. Guidance for planning and implementing catch-up vaccination. Geneva: WHO, 2021. .
10. World Health Organization (WHO). Immunization Agenda 2030: A Global Strategy to Leave No One Behind. 2020.
11. Noori T, Hargreaves S, Greenaway C, van der Werf M, Driedger M, Morton RL, et al. Strengthening screening for infectious diseases and vaccination among migrants in Europe: What is needed to close the implementation gaps? Travel Med Infect Dis. 2021;39:101715.
12. European Centre for Disease Prevention and Control. Public health guidance on screening and vaccination of migrants in the EU/EEA. Available from: https://www.ecdc.europa.eu/en/publications-data/public-health-guidance-screening-and-vaccination-infectious-diseases-newly. ECDC. Sweden: Stockholm; 2018.
13. Public Health England. Vaccination of individuals with uncertain or incomplete immunisation status. Updated 26th August 2021. Available from: https://www.gov.uk/government/publications/vaccination-of-individuals-with-uncertain-or-incomplete-immunisation-status/vaccination-of-individuals-with-uncertain-or-incomplete-immunisation-status [Accessed 21st September 2021].
14. Morse JM (1995) The Significance of Saturation. Qual Health Res 5(2): 147-149 Available from: [https://journals.sagepub.com/doi/10.1177/104973239500500201]
.
15. Braun V, Clarke V. Thematic analysis. In: Cooper H, Camic PM, Long DL, editors. APA handbook of research methods in psychology, 2: research designs: quantitative, qualitative, neuropsychological, and biological. Washington, DC: American Psychological Association; 2012.
16. AuYOung M, et al. Addressing racial/ethnic inequities in vaccine hesitancy and uptake: lessons learned from the California alliance against COVID-19. J Behavioural Med 2022; 22nd Jan https://link.springer.com/article/10.1007/s10865-022-00284-8.
17. Socha A, Klein J. What are the challenges in the vaccination of migrants in Norway from healthcare provider perspectives? A qualitative, phenomenological study
BMJ Open 2020;10:e040974. doi: 10.1136/bmjopen-2020-040974
.
18. Moussaoui S, Aurousseau AM, Nappez S, Cornaglia J, Delobre G, Blanchi S, et al. Immunization Catch-Up for Newly Arrived Migrants in France: A Cross-Sectional Study among French General Practitioners. 2021;9(6):21.
19. Ravensbergen SJ, et al. National approaches to the vaccination of recently arrived migrants in Europe: a comparative policy analysis across 32 European countries. Trav Med and Infect Dis 2019; 33-38. .
20. Giambi C, Del Manso M, Marchetti G, Olsson K, Adel Ali K, Declich S, et al. Immunisation of migrants in EU/EEA countries: Policies and practices. Vaccine. 2019;37(36):5439-51.
21. Evlampidou I, et al. Low hepatitis B testing among migrants: a cross-sectional study in a UK city. Br J Gen Pract 2016; 66)647): e382-e391. .
22. Crawshaw AF, Deal A, Rustage K, Forster AS, Campos-Matos I, Vandrevala T, et al. What must be done to tackle vaccine hesitancy and barriers to COVID-19 vaccination in migrants? Journal of Travel Medicine. 2021;28(4):01.

For peer review only - http://bmjopen.bmj.com/site/about/guidelines.xhtml

23. Jama A, et al. Perspectives on the measles, mumps and rubella vaccination among Somali Mothers in Stockholm. Int J Environmental Research and Public Health 2018; 15: 11.https://www.mdpi.com/1660-4601/15/11/2428.

24. Kasstan B. Vaccines and vitriol: an anthropological commentary on vaccine hesitancy, decision-making and interventionism among religious minorities. Anthrop & Med 2020; 13 Nov: 411-419 https://www.tandfonline.com/doi/full/10.1080/13648470.2020.1825618?casa_token=DiweSjBRwMQAAAAA%3Aw1JFAzqfbT8KsQTnz72bbpGy7pcmgRjehDA8OeGAMmOD1esZFPHL0IWhxq2M-p0vU5Z4F3-cFEaZTQ.

25. Larson HJ, et al. Understanding vaccine hesitancy around vaccines and vaccination from a global perspective: a systematic review of published literature, 2007-2012. Vaccine; 32: 19: 2150-2159.

26. WHO. Immunization coverage. July 15, 2021. Available from: https://www.who.int/news-room/fact-sheets/detail/immunization-coverage.

27. Tankwanchi AS, Bowman B, Garrison M, Larson H, Wiysonge CS. Vaccine hesitancy in migrant communities: a rapid review of latest evidence. Curr Opin Immunol. 2021;71:62-8.

28. Doctors of the World UK. Vaccine Confidence Toolkit. England: London.2021. [Available from: https://www.doctorsoftheworld.org.uk/what-we-stand-for/supporting-medics/vaccine-confidence-toolkit/

29. Hui, C., Dunn, J., Morton, R., Staub, L. P., Tran, A., Hargreaves, S., Greenaway, C., Biggs, B. A., Christensen, R., & Pottie, K. (2018). Interventions to Improve Vaccination Uptake and Cost Effectiveness of Vaccination Strategies in Newly Arrived Migrants in the EU/EEA: A Systematic Review. International journal of environmental research and public health, 15(10), 2065. https://doi.org/10.3390/ijerph15102065.

For peer review only - http://bmjopen.bmj.com/site/about/guidelines.xhtml

Standards for Reporting Qualitative Research (SRQR)*

http://www.equator-network.org/reporting-guidelines/srqr/

Title and abstract

	Page/line no(s).
**Title** - Concise description of the nature and topic of the study Identifying the study as qualitative or indicating the approach (e.g., ethnography, grounded theory) or data collection methods (e.g., interview, focus group) is recommended	1
**Abstract** - Summary of key elements of the study using the abstract format of the intended publication; typically includes background, purpose, methods, results, and conclusions	2

Introduction

**Problem formulation** - Description and significance of the problem/phenomenon studied; review of relevant theory and empirical work; problem statement	6-7
**Purpose or research question** - Purpose of the study and specific objectives or questions	6-7

Methods

**Qualitative approach and research paradigm** - Qualitative approach (e.g., ethnography, grounded theory, case study, phenomenology, narrative research) and guiding theory if appropriate; identifying the research paradigm (e.g., postpositivist, constructivist/ interpretivist) is also recommended; rationale**	8-10
**Researcher characteristics and reflexivity** - Researchers' characteristics that may influence the research, including personal attributes, qualifications/experience, relationship with participants, assumptions, and/or presuppositions; potential or actual interaction between researchers' characteristics and the research questions, approach, methods, results, and/or transferability	10
**Context** - Setting/site and salient contextual factors; rationale**	9
**Sampling strategy** - How and why research participants, documents, or events were selected; criteria for deciding when no further sampling was necessary (e.g., sampling saturation); rationale**	10
**Ethical issues pertaining to human subjects** - Documentation of approval by an appropriate ethics review board and participant consent, or explanation for lack thereof; other confidentiality and data security issues	8
**Data collection methods** - Types of data collected; details of data collection procedures including (as appropriate) start and stop dates of data collection and analysis, iterative process, triangulation of sources/methods, and modification of procedures in response to evolving study findings; rationale**	10

For peer review only

For peer review only - http://bmjopen.bmj.com/site/about/guidelines.xhtml

**Data collection instruments and technologies** - Description of instruments (e.g., interview guides, questionnaires) and devices (e.g., audio recorders) used for data collection; if/how the instrument(s) changed over the course of the study	10
**Units of study** - Number and relevant characteristics of participants, documents, or events included in the study; level of participation (could be reported in results)	11
**Data processing** - Methods for processing data prior to and during analysis, including transcription, data entry, data management and security, verification of data integrity, data coding, and anonymization/de-identification of excerpts	10
**Data analysis** - Process by which inferences, themes, etc., were identified and developed, including the researchers involved in data analysis; usually references a specific paradigm or approach; rationale**	10
**Techniques to enhance trustworthiness** - Techniques to enhance trustworthiness and credibility of data analysis (e.g., member checking, audit trail, triangulation); rationale**	10

Results/findings

**Synthesis and interpretation** - Main findings (e.g., interpretations, inferences, and themes); might include development of a theory or model, or integration with prior research or theory	11-22
**Links to empirical data** - Evidence (e.g., quotes, field notes, text excerpts, photographs) to substantiate analytic findings	11-22

Discussion

**Integration with prior work, implications, transferability, and contribution(s) to the field** - Short summary of main findings; explanation of how findings and conclusions connect to, support, elaborate on, or challenge conclusions of earlier scholarship; discussion of scope of application/generalizability; identification of unique contribution(s) to scholarship in a discipline or field	23-25
**Limitations** - Trustworthiness and limitations of findings	23-24

Other

**Conflicts of interest** - Potential sources of influence or perceived influence on study conduct and conclusions; how these were managed	26
**Funding** - Sources of funding and other support; role of funders in data collection, interpretation, and reporting	26

*The authors created the SRQR by searching the literature to identify guidelines, reporting standards, and critical appraisal criteria for qualitative research; reviewing the reference lists of retrieved sources; and contacting experts to gain feedback. The SRQR aims to improve the transparency of all aspects of qualitative research by providing clear standards for reporting qualitative research.

For peer review only - http://bmjopen.bmj.com/site/about/guidelines.xhtml

**The rationale should briefly discuss the justification for choosing that theory, approach, method, or technique rather than other options available, the assumptions and limitations implicit in those choices, and how those choices influence study conclusions and transferability. As appropriate, the rationale for several items might be discussed together.

Reference:

O'Brien BC, Harris IB, Beckman TJ, Reed DA, Cook DA. **Standards for reporting qualitative research: a synthesis of recommendations.** *Academic Medicine*, Vol. 89, No. 9 / Sept 2014 DOI: 10.1097/ACM.0000000000000388

For peer review only

BMJ Open

"We don’t routinely check vaccination background in adults”: A national qualitative study of barriers and facilitators to vaccine delivery and uptake in adult migrants through UK primary care

Journal:	*BMJ Open*
Manuscript ID	bmjopen-2022-062894.R1
Article Type:	Original research
Date Submitted by the Author:	01-Sep-2022
Complete List of Authors:	Carter, Jessica; St George's University of London, Institute for Infection and Immunity<br>Mehrotra, Anushka; St George's University of London, Institute for Infection and Immunity<br>Knights, Felicity; St George's University of London, Institute for Infection and Immunity<br>Deal, Anna; St George's University of London, Institute for Infection and Immunity; LSHTM<br>Crawshaw, Alison; St George's University of London, Institute for Infection and Immunity<br>Farah, Yasmin; St George's University of London, Institute for Infection and Immunity<br>Goldsmith, Lucy; St George's University of London, Infection and Immunity Research Institute, and Population Health Research Institute<br>Wurie, Fatima; UKHSA<br>Ciftci, Yusuf; Doctors of the World UK<br>Majeed, Azeem; Imperial College London, Primary Care<br>Hargreaves, Sally; St George's University of London, Institute for Infection and Immunity
**Primary Subject Heading**:	Infectious diseases
Secondary Subject Heading:	General practice / Family practice, Infectious diseases
Keywords:	Infection control < INFECTIOUS DISEASES, Public health < INFECTIOUS DISEASES, Health policy < HEALTH SERVICES ADMINISTRATION & MANAGEMENT

SCHOLARONE™
Manuscripts

I, the Submitting Author has the right to grant and does grant on behalf of all authors of the Work (as defined in the below author licence), an exclusive licence and/or a non-exclusive licence for contributions from authors who are: i) UK Crown employees; ii) where BMJ has agreed a CC-BY licence shall apply, and/or iii) in accordance with the terms applicable for US Federal Government officers or employees acting as part of their official duties; on a worldwide, perpetual, irrevocable, royalty-free basis to BMJ Publishing Group Ltd ("BMJ") its licensees and where the relevant Journal is co-owned by BMJ to the co-owners of the Journal, to publish the Work in this journal and any other BMJ products and to exploit all rights, as set out in our licence.

The Submitting Author accepts and understands that any supply made under these terms is made by BMJ to the Submitting Author unless you are acting as an employee on behalf of your employer or a postgraduate student of an affiliated institution which is paying any applicable article publishing charge ("APC") for Open Access articles. Where the Submitting Author wishes to make the Work available on an Open Access basis (and intends to pay the relevant APC), the terms of reuse of such Open Access shall be governed by a Creative Commons licence – details of these licences and which Creative Commons licence will apply to this Work are set out in our licence referred to above.

Other than as permitted in any relevant BMJ Author's Self Archiving Policies, I confirm this Work has not been accepted for publication elsewhere, is not being considered for publication elsewhere and does not duplicate material already published. I confirm all authors consent to publication of this Work and authorise the granting of this licence.

For peer review only

"*We don't routinely check vaccination background in adults*": A national qualitative study of barriers and facilitators to vaccine delivery and uptake in adult migrants through UK primary care

Authors

*Jessica Carter[1] -MBBS jcarter@sgul.ac.uk ORCID 0000-0001-9590-3146

*Anushka Mehrotra[1] MBBS GP anushka.mehrotra@nhs.net

*Felicity Knights[1]- MBBS, MA (Cantab), MPH fknights@sgul.ac.uk ORCID 0000-0002-3007-1438

Anna Deal[1,2] -BSc, MSc, adeal@sgul.ac.uk ORCID: 0000-0001-6168-6542

Alison F Crawshaw[1] – MSc MA Hons. (Oxon), acrawsha@sgul.ac.uk, Orcid ID: 0000-0003-0450-7258

Yasmin Farah BSc[1]

Lucy P Goldsmith – PhD[1]

Fatima Wurie[3]

Yusuf Ciftci[4] PhD

Azeem Majeed[5] a.majeed@imperial.ac.uk, Orcid ID 0000-0002-2357-9858

Sally Hargreaves[1] - PhD FRCPE s.hargreaves@sgul.ac.uk Orcid ID: 0000-0003-2974-4348

***Joint first authors**

[1] The Migrant Health Research Group, Institute for Infection and Immunity, St George's, University of London, London, UK

[2]Faculty of Public Health and Policy, London School of Hygiene and Tropical Medicine, London

[3]Office for Health Improvement and Disparities, Department of Health and Social Care; UK Health Security Agency, London, UK (F Wurie)

[4]Doctors of the World, UK (Y Ciftci)

[5]Department of Primary Care and Public Health, Imperial College London, Charing Cross Campus, The Reynolds Building, St Dunstan's Road, London, W6 8RP, UK (A Majeed)

For peer review only - http://bmjopen.bmj.com/site/about/guidelines.xhtml

Correspondence to: Dr Sally Hargreaves, The Migrant Health Research Group, Institute for Infection and Immunity, St George's, University of London, London, UK s.hargreaves@sgul.ac.uk

For peer review only

For peer review only - http://bmjopen.bmj.com/site/about/guidelines.xhtml

Abstract

- **Objectives:** Explore primary care professionals views around barriers/facilitators to catch-up vaccination in adult migrants(foreign-born; over 18-years) with incomplete/uncertain vaccination status and for routine vaccines to inform development of interventions to improve vaccine-uptake and coverage.
- **Design:** Qualitative interview study with purposive sampling and thematic analysis
- **Setting:** UK primary-care
- **Participants:** 64 primary care professionals (PCPs): 48 clinical-staff including GPs, Practice Nurses, healthcare assistants (HCAs); 16 administrative-staff including practice managers and receptionists (mean age 45 years; 84.4% female; a range of ethnicities).
- **Results:** Participants highlighted direct and indirect barriers to catch-up vaccines in adult migrants who may have missed vaccines as children, missed boosters, and not be aligned with the UK's vaccine schedule, from both personal and service-delivery levels, with themes including: lack of training and knowledge of guidance among staff; unclear or incomplete vaccine records; and lack of incentivization (including financial) and dedicated time and care pathways. Adult migrants were reported as being excluded from many vaccination initiatives, most of which focus exclusively on children. Where delivery models existed, they were diverse and fragmented but included a combination of opportunistic and proactive programmes. PCPs noted that migrants expressed to them a range of views around vaccines, from positivity to uncertainty, to refusal, with specific nationality groups reported as more hesitant about specific vaccines, including MMR.
- **Conclusions:** WHO's new Immunization Agenda (IA2030) called for greater focus to be placed on delivering vaccination across the life-course, targeting under-immunised groups for catch-up vaccination at any age, UK primary care services therefore have a key role. Vaccine uptake in adult migrants could be improved through implementing new financial incentives or inclusion of adult migrant vaccination targets in QOF, strengthening care pathways and training, and working directly with local community-groups to improve understanding around the benefits of vaccination at all ages.

Keywords: Vaccination, catch-up vaccination, migrant, health policy, COVID-19

For peer review only

Article summary

Strengths and limitations

(i) A key strength of the study is the number and variety of primary care staff included from across England in diverse settings

(ii) Interviewees were a self-selecting group, which may have affected the profile of those responding – a common consideration in qualitative research.

(iii) A large number of practices were involved, however, and this diversity and the scale of the study is likely to have added to the breadth of experience and solutions reflected in our findings, as well as enhancing the validity.

(iv) The structure and experience of primary care across Europe and between the devolved nations of the UK may differ so the recruitment only within England may limit the generalisability of the findings, however we note other European and international studies have come to the similar conclusions.

Funding statement

This work has been funded by the NIHR (NIHR300072). JC is funded by an NIHR-in practice clinical fellowship (NIHR300290). SH, AFC and LPG are funded by the NIHR (NIHR300072), and SH and AFC are additionally funded by the Academy of Medical Sciences (SBF005\1111). SH acknowledges funding from the Novo Nordisk Foundation/La Caixa Foundation (Mobility– Global Medicine and Health Research grant), the World Health Organization, and the MRC. AD is funded by the MRC (MR/N013638/1). AM is supported by the NIHR Applied Research Collaboration NW London. FK is supported by a Health Education England/NIHR Academic Clinical Fellowship. We acknowledge the support of the European Society of Clinical Microbiology and Infectious Diseases (ESCMID) Study Group for Infections in Travellers and Migrants (ESGITM). FW is a member of the Vulnerable Migrants Wellbeing Project Advisory Board, led by the University of Birmingham and Doctors of the World and funded by the Nuffield Foundation. The funders did not have any direct role in the writing or decision to submit this manuscript for publication. The views expressed are those of the author(s) and not necessarily those of the NHS, the NIHR, or the Department of Health and Social

For peer review only

Care. The funder of the study had no role in study design, data collection, data analysis, data interpretation, or writing of the report.

Competing interest statement

All authors report having nothing to declare.

Data availability statement

All data relevant to the study are included in the article or uploaded as supplementary information.

Acknowledgements

We thank members of our NIHR Patient and Public Involvement Project Advisory Board (Babatunde Tikare, Larysa Agbaso, Monika Hartmann, Saliha Majeed) and additional members of our Project Board for valuable contributions.

For peer review only

Introduction

Adult migrants in Europe – particularly those from low- and middle-income countries – may be at risk of under-immunisation for routine vaccinations due to missed vaccines and doses as children (due to lack of availability, war/disruption, poorly functioning health systems, and personal, social, and physical barriers to accessing vaccines), and/or missed boosters, and differing vaccination schedules in their home country (especially for newer vaccines such as HPV), and so may not be aligned with the UK's vaccination schedule on arrival (1-3). Additional vaccines may be recommended if they return to their home country, or for specific occupations (eg, tetanus and hepatitis B). Some migrant populations are known to be at risk of under-immunisation (2, 4-6) and were involved in recent outbreaks of vaccine-preventable diseases in Europe, including measles (1). However, adolescent and adult migrants, beyond school age, are often not routinely incorporated into vaccination programmes on arrival to most European countries, including the UK (7). The COVID-19 pandemic has highlighted shortfalls in engaging older migrants, and other marginalised groups, in vaccination programmes (8), yet it has also presented a range of new opportunities and innovations in vaccine service delivery and policy making to these groups, which merit greater consideration beyond the pandemic.

The World Health Organization's new Immunization Agenda 2030 (IA2030) (9) aims to improve vaccine coverage for vaccine-preventable diseases (VPDs), placing an emphasis on achieving equitable access for vulnerable populations and integrating catch-up vaccination for missed vaccines and doses throughout the life-course. WHO recommends that all countries have a catch-up vaccination policy and catch-up vaccination schedule in place, to close immunisation gaps that would otherwise compound as populations increase in age (10, 11), and that it is always "better to vaccinate late than never". Although age limits apply for administration of a small number of vaccines, for most VPDs, providing vaccines late will still result in protection against morbidity and mortality, as well as reducing transmission and risk of outbreaks, with personal and community-level benefits. Specific WHO guidance for catch-up vaccination is available (10); in Europe the European Centre for Disease Prevention and Control (ECDC) has published guidance on catch-up vaccination in children and adult migrants on arrival (12, 13), calling for healthcare providers to consider revaccinating adult migrants with uncertain vaccination status or no recorded history of

For peer review only

vaccination. For UK arrivals, advice is available from the UKHSA on the 'vaccination of individuals with uncertain or incomplete immunisation status' (see Panel 1), which will be relevant to most arriving migrants (14). However, the extent to which these guidelines and policies are put into practice and prioritised by UK primary care – tasked with delivering the majority of the UK's vaccine programmes – is not known. No studies to date have explored the views and experiences of front-line primary care teams on approaches to catch-up adult vaccination in arriving migrants. We therefore did a national qualitative in-depth interview study with a range of primary care professionals to understand the challenges and needs of migrant populations with regards to catch-up vaccinations programmes, and facilitators and solutions to addressing gaps in service provision.

Panel 1: Vaccination of individuals with uncertain or incomplete immunisation status. Reproduced from (14).

From tenth birthday onwards:

- Td/IPV and MenACWY*and MMR
  Four week gap
  Td/IPV and MMR
  Four week gap
  Td/IPV

First booster of Td/IPV – preferably 5 years following completion of primary course. Second booster of Td/IPV - ideally 10 years (minimum 5 years) following first booster.

- HPV:

-all females who have been eligible remain so up to their 25th birthday

-males born on or after 01/09/2006 are eligible up to their 25th birthday

- Subsequent vaccination – as per UK schedule (see Flu Vaccine, Shingles vaccine, PPV) and COVID-19.

**Those aged from 10 years up to 25 years who have never received a MenC-containing vaccine should be offered MenACWY. Those aged 10 years up to 25 years may be eligible or may shortly become eligible for MenACWY usually given around 14 years of age. Those born on/after 01/09/1996 remain eligible for MenACWY until their 25th birthday.*

For peer review only

Methods

Design

Qualitative semi-structured interviews of both clinical and administrative staff were undertaken by telephone, following a topic guide collaboratively developed by the research team with support from a board of migrant representatives. The guide was piloted prior to data collection and iteratively developed throughout the data collection process, with the addition of further prompts and probes to develop richer understanding and addressed key areas around approach to vaccination of adult migrants, factors affecting vaccine hesitancy and uptake, and possible interventions to strengthen delivery (Panel 2). Ethics approval was granted by the Health Research Authority (reference number: REC 20/HRA/1674). The team comprised two GPs and four academics, and was supported by a wider project board of a diverse group of migrant ambassadors. The range of professional and personal experience supported integration of multiple perspectives throughout the design, collection and analysis stages. The inclusion of two GPs in the research team brought knowledge of UK primary care to the study but required careful reflection during interviews and data analysis and was balanced by the inclusion of non-GP research team members at the interview stage.

Patient and public involvement

A board of migrant representatives supported the design of this study and development of the topic guides.

For peer review only

Panel 2: Topic guide

Background Questions:
- Proportion of migrants seen at practice, migrant health training and experience
- General barriers and facilitators to registration and provision of care for migrant patients

Questions pertaining to Vaccination of Adult Migrants:
- Are you aware of any guidance regarding vaccination and infectious disease screening in migrants?
- Have there been any outbreaks of vaccine preventable diseases or cases of vaccine preventable diseases in your area involving migrants – we are particularly interested in adults? (If yes, what do they think the reasons might be?
- What experience have you had with adult migrant patients and vaccination?

Questions regarding Practice Approach to Vaccination of Adult Migrants:
- How do you approach catch-up vaccination in the adult migrant patient group, specifically ensuring adult migrants are caught up to align with the UK schedule?
- Who is responsible for vaccination at your practice?
- Is there a mechanism at your practice or in your area to engage adult migrants on catch-up vaccination?
- Is there a local catch-up vaccination pathway?
- Do you target any specific groups?

Questions regarding possible interventions to increase uptake of catch-up vaccination in migrants:
- If there are no mechanisms/pathways in place locally do you think there should be?
- What could such a system look like?
- Are you aware of any other interventions relating to vaccination in migrants? If so, what made them successful/ unsuccessful?
- What do you think about a migrant health check, and what vaccinations would be important to cover in this for adult migrants in your view?
- What in your opinion would be the key to a successful intervention/ behaviour change in primary care?

Setting

Latest figures show there are 6822 GP practices in England, the majority are in urban environments as are migrant populations. Participants were recruited from 50 GP practices. 50 (78%) participants were from practices in urban settings and 14 participants (22%) from suburban or rural settings across England. Practices were based in one of six local clinical research networks (CRNs) — CRN Kent, Surrey and Sussex; CRN South London; CRN North Thames; CRN North West London; CRN West Midlands; and CRN Greater Manchester with the exception of a practice in Newcastle and another in Oxford.

For peer review only

Participants

Participants were purposively sampled to capture the diversity of experiences in general practice, from administrative and clinical primary care roles, and practices which varied both in size, and urban/rural location, factors which could influence the number of migrant patients and the organisation of care. Recruitment occurred via local Clinical Research Networks, 'word of mouth' invitations from colleagues and a number of primary care newsletters, social media groups and practice manager mailing lists. All participants who expressed an interest in taking part were e-mailed a participant information sheet and consent form and invited to a telephone interview at a time of mutual convenience, with written informed consent being given in advance. £20 vouchers were given as compensation for each participant's time.

Data collection

Telephone interviews, between 30-60 minutes, were carried out by JC (GP) FK, (GP registrar) and AD and AFC (academic researchers) who made field notes in the majority of cases. Interviews were distributed randomly to research team members. Findings from the initial interviews were discussed across the group and led to the development of additional prompts and lines of questioning in the topic guide, as well as additional lines of questioning for non-clinical participants. All but three of the interviews were digitally recorded and transcribed verbatim by professional transcription service. The remaining three were lost through technical error but were typed up from extensive field notes. Transcripts were anonymised with a coded participant number and checked for accuracy. Data collection continued until there was thematic saturation (15) across all core themes as unanimously agreed across the team.

Data Analysis

Data analysis was inductive, based on the stages of thematic analysis (16). The transcripts were read repeatedly by AM (familiarisation) and emerging themes and patterns were identified and discussed with FK and JC who had also previously immersed themselves in the data. Initially, a coding of 10 transcripts on Microsoft Excel by AM allowed identification of emergent themes and discussion with FK and JC. NVivo (version 13) was then used to organise codes and iteratively refine and develop the emerging coding framework through a process of constant comparison, with close attention paid to non-confirmatory cases which contradicted existing themes. The final coding and

For peer review only - http://bmjopen.bmj.com/site/about/guidelines.xhtml

themes were conceptualised through recurrent discussion by AM, FK, JC and SH. Active reflexivity was attempted from the study's onset, and input from across the multidisciplinary team, with support from the migrant advisory board, facilitated robust discussion throughout.

Results

In total, 64 interviews were conducted. 48 interviews were held with primary care staff: 25 GPs, 15 practice nurses, seven healthcare assistants (HCAs), one clinical pharmacist, 11 practice managers, and five receptionists. Participants were aged between 25 and 74 with a mean age of 45 years old (SD 11.8) and had been working in primary care between 1 and 35 years (mean 12.27 years SD 9.45). The majority of staff (50 [78.1%]) worked in urban practices. Characteristics of included participants are presented in Table 1.

Table 1: Characteristics of participants.

Characteristics	Total participants (n=64)
Staff type	General Practitioners (GPs): 25 (39%)<br>Practice Nurses (PNs): 15 (23.5%)<br>Healthcare assistants (HCAs): 7 (11%)<br>Pharmacist: n=1<br>Practice Managers: 11 (17%)<br>Receptionists: 5 (8%)
Ethnicity	African: 4 (6.3%)<br>Other Asian background: 2 (3.1%)<br>Mixed: 3 (4.7%)<br>Other white: 5 (7.8%)<br>Caribbean: 1 (1.6%)<br>Indian: 11 (17.2%)<br>Pakistani: 3 (4.7%)<br>White British: 32 (50%)<br>White Irish: 2 (4.7%)
Age	45 years (SD 11.8 years)
Sex	Female: 54 (84.4%)<br>Male: 10 (15. 6%)

For peer review only - http://bmjopen.bmj.com/site/about/guidelines.xhtml

Participants had varied exposure of vaccine delivery in migrant patients, but the data were convergent across this breadth of migrant healthcare experience, geographical area, and participant profession. The main themes that emerged from data analysis were; the existence of multiple barriers to the delivery of catch-up vaccination to migrant patients, including vaccine acceptance and PCP training,; the fragmented nature of adult migrant catch-up vaccination models despite existence of guidelines; the role of travel vaccination and occupational health have in adult migrant catch-up vaccination and next steps for strengthening delivery of catch up vaccination with existence of positive attitudes to strengthening primary care's role through numerous PCP enacted or suggested solutions to barriers given.

Existence of multiple barriers reported by PCPs to vaccine uptake in adult migrants

<u>Patient acceptance of vaccines from PCPs</u>

Participants reported that their migrant patients express a range of views around vaccines from positivity to uncertainty, to refusal. Generalised mistrust and misinformation about vaccinations in migrant groups was commonly reported, which was often perceived by PCPs as resistance to information-sharing about the vaccine in question.

"It's really hard to break through that barrier of… this is the evidence [about this vaccine]… I don't think they're listening… they're thinking… this is someone from my community saying this [other information]. And you're not from my community… I don't know if you have the best interests [in mind]." GP10

<u>Different nationalities have different views on vaccines</u>

Some PCPs gave their views on vaccine acceptance and uptake linked to specific nationalities, and most often reported beliefs or experiences that migrants originating from Eastern Europe, France and Italy, Somalia and Bangladesh tend to be hesitant about vaccines. Table 2 provides illustrative quotes. Fixed negative views around vaccines were most often reported from Eastern European migrants, who were also viewed as having poor vaccination records and as wanting to follow a different vaccination schedule (as per protocols in their home country), with some returning to their own countries to be vaccinated. The doctor-patient relationship was highlighted as a key

For peer review only

factor in tackling mistrust and vaccine hesitancy; some PCPs felt this represented a barrier and that it was easier for migrant patients to connect with PCPs from their own communities.

Table 2: Perceptions of staff around acceptance and uptake in specific nationality groups

Participant reporting	Quote
GP	…now it's more Bangladeshi, so Somalian was really with the MMR thing. But we still find more Bangladeshi families delaying or refusing the immunisation of their babies……So, yes they always blame… This is too much, the baby is young, we're not sure about the long term effects.
GP	"[The Somalian population]…is a massive concern for us, with regards the patients unfortunately, falsely attributing MMR with an autism link"……." I think it was the belief of autism, but why more in the Somali community than any other minority group, I'm not too sure."
HCA	[The Somalian population are] … very happy to vaccinate as elderly patients. But, [they think]…the children will get something, get over it. And I think with MMR, they do feel that there's side effects. They think that it causes Autism and things like that.
Practice nurse	I don't know where, Somalia or Eritrea that there was only one interpreter in London who could speak their language. Even their care worker obviously could not speak their language. And so, trying to get immunisation history or any history out of these two young men was totally impossible
HCA	I would say that Europeans [migrants], they refuse because they think they've had them, even if it's been a long time and they don't know.
GP	What I have noticed is that when a patient comes from… Eastern European countries… they do come in with a vaccination record. It's usually incomplete… and sometimes we doubt [it is true and], whether…you can pay someone to give you a vaccination record but it actually hasn't happened.

Language barriers

Language barriers leading to an inability to communicate vaccination histories and understand vaccine offers were felt by participants to reduce the likelihood of migrants accessing catch-up vaccinations, compounded by a lack of written communication in variety of languages about vaccine services.

"Language can be a barrier for subtleties of communication, despite language line" GP21

For peer review only - http://bmjopen.bmj.com/site/about/guidelines.xhtml

"I think we probably ought to translate that communication [about vaccine programmes] in written Bengali, and perhaps Somali as well." GP10

"There's usually a long wait and possibly a language barrier as well that may stop [people] from communicating or trying to make that appointment" PN 15

Lack of accurate vaccine histories and fear of immigration

Participants raised the fact that unclear or poorly documented vaccination histories meant staff were unclear as to what to do, as well as highlighting problems with vaccination records not being transferred within the NHS, and a lack of availability of records from migrants' home countries, including limited translation of previous records into English. Some migrants were reported as having different ages recorded, leading to challenges determining vaccine eligibility. Issues were raised about immigration status and PCPs reported migrant patient fears about being reported to authorities if migrant patients attended the GP and disclosed country of origin as part of their vaccine history.

"And we're certainly not being given any records from other countries that might support [vaccination catch-up]… unless the patient is super well-organised and providing that it happens to be in English or a language that's directly transferrable…" Admin 6

"I think immigration status, out of anything, is going to be the main issue. A lot of people that live in this country without status, going to the GP is a massive risk." PN 13

Lack of training and unawareness of guidelines amongst PCPs

Health-system and staff barriers to providing catch-up vaccination for adult migrants included lack of training among staff and lack knowledge of guidance around catch-up vaccination.

"The nurses would need some kind of education in how to complete incomplete vaccination programmes in adults" Admin 12

"So, no, I'm not aware of any guidance for [vaccination in] migrant people" GP 2

Time and financial pressures

For peer review only

There were also a number of additional barriers to accessing care at staff and system level which were felt to reduce the likelihood of adult migrant patients being offered and accepting a catch-up vaccine or travel vaccines through the travel clinic. These included a lack of time to carry out proactive catch-up programmes, or to follow up on opportunistic or challenging conversations where a vaccination need was highlighted, especially when using a translator. The financial pressures and impact of vaccination programmes falling outside of current incentive schemes, such as quality outcomes framework (QOF), also impacted on the time available for the programmes.

"It's just time pressure, the way that the general practice is working at the moment unfortunately is reactive…And so, with things like vaccinations, especially if it's catch up or screening, can always wait… [because] you're going to deal with [someone's chest infection or…diabetes] before you deal with their symptomatic screening. GP6

There are "no incentives for catchup vaccination, MMR… especially compared to childhood immunisations and chronic diseases in QOF." (GP16)

The above represent barriers across all vaccinations. There were barriers reported to specific vaccines in the UK schedule and these have been summarized by vaccine in table 3.

Table 3: Key barriers to adults acquiring specific vaccines

Disease	Key barrier perceived by health care professionals	Quote	Professional
**Influenza**	Multiple staff involved creates risk of disjointed process	'Reception staff call…give [clinicians] the list…then the nurses…[and] doctors vaccine them for both child …and adult.'	Admin 13
	Perceived side effects	'[Adults with flu jab] sometimes they don't want it because they said they had it before and they had side effects, so yes, that's the main thing.'	GP 13
	Perceived poor understanding of flu amongst migrant patients	'I find it difficult to convince them that [flu vaccine]… is useful. Because most …[adult migrants] don't understand the concept of flu.'	GP 4

For peer review only - http://bmjopen.bmj.com/site/about/guidelines.xhtml

Disease	Key barrier perceived by health care professionals	Quote	Professional
	Low uptake amongst younger adult migrant patients	'We find that, generally, the over 65s will take it and under 65s will have very low uptake…[not sure] it makes a difference with what ethnic background they're from…'	GP 16
	Specific health beliefs surrounding flu vaccine and immune system	'[Adult migrants]… are refusing because they want to have a[immune] system and teach their body to fight against a virus…[or] they had bad side effects'	HCA 4
**Hepatitis A**	Requires patient to proactively seek vaccine	'We do …[this] when a patient contacts us, because they're either worried about hepatitis or they're thinking they're going to travel [to their home country]'	Admin 13
**Hepatitis B**	Not within NHS catch up vaccination schedule	'Vaccination is not within the schedule, so it has to be treated like a private prescription...and [can be] occupation[al] [eg nurses from South India]'	GP 4
**HPV**	Taboo subject for some migrants	'Doesn't seem to be a very good uptake of it, in the migrant communities that we have…I think an anything remotely to do, within the genitalia area. When you to to discuss that… it's normally a difficult conversation to have with a lot of the migrant families…. I think they find that a bit of a taboo subject… [they] generally come in groups… [with] mum, dad and maybe a couple of children… [which] makes conversations like HPV… more difficult to discuss.'	HCA 6
**Meningococcal**	Potential for missed opportunities outside of travel related risk	'If [adult migrants have] …not had a meningitis, I will always offer that to them as part of the travel thing'	PN 2
**MMR**	False link with autism	'The Somalian population… falsely attributing MMR with an autism link….'	GP 1

For peer review only

Disease	Key barrier perceived by health care professionals	Quote	Professional
	Electronic systems provide excess alerts leading to health care professional desensitisation	'EMIS (an online practice system) is very annoying because every single patient for who it doesn't have MMR date, it says MMR is outstanding…when people have come, especially if they're refugees or asylum seekers, they won't have that paperwork'	GP 2
	Less perception of need at older age	'[When migrant patients register]…Especially for under 40, we try to find out, the MMRs, if they have them…If they are young they will accept. But then, the standard for patients over 40, they don't want to anymore.'	HCA 4
**Shingles**	Lack of understanding of shingles	'There's not enough education around it.. it's not something as well-known as, say the flu…it's difficult to get [a translator] for every single patient, to educate them what shingles is..so, I think… it's more education that's needed.'	HCA 6

Fragmented models for vaccine delivery to adult migrants

Almost all clinical staff reported the availability of good catch-up programmes for childhood vaccination among recently arrived migrants, with some PNs specifically quoting the Public Health England Schedule for individuals with uncertain vaccination status. Incentivization for under-5s vaccination included the Quality Outcome Framework (QOF), and well-resourced systems to ensure children are not missed, including the vaccination record 'red book' and using recall systems to contact patients, such as sending reminder texts. By contrast, adult migrants were often reported as being excluded from vaccination initiatives. One GP stated that over 5's and adults are sometimes assumed to be *"up to date from the country they come from"*, and many staff, especially GPs and administrators, were not aware of any catch-up vaccination programmes for adult migrants.

For peer review only - http://bmjopen.bmj.com/site/about/guidelines.xhtml

"We don't routinely check vaccination background in adults" GP 16

"We do catch-up vaccinations for children and young adults who've missed their primary vaccinations, but in terms of adults or people who are arriving to the UK, no" Admin 6

"Ad hoc. We haven't had a particular programme for [adult catch up vaccinations] GP15

Where adult catch-up vaccination was provided, models of delivery were diverse and fragmented, comprising a range of clinics and providers, different staff members (primarily nurses), and a combination of opportunistic and proactive programmes. Providers of catch-up vaccination for adult migrants included: NHS GP practices, detention centres (for undocumented migrants and asylum seekers), migrant-specific or language-specific clinics, private clinics and specialised clinics (e.g. sexual health clinic in China Town), with distinct benefits and challenges.

Detention centres: "Interpreters weren't always readily provided when I was at the detention centres. We found that really difficult and it took several visits [to determine which vaccines were required and these to be given]" PN13

"[on local community infectious disease led clinic] *And they have a large Somalian support network there, so they have interpreters, and bits and pieces………. They will go in, and there will be a Somalian phlebotomist and doctor, and so they engage with it that way, much easier." HCA 6*

Respondents reported vaccinations programmes were a mix of opportunistic and proactive delivery approaches. Proactive programmes included methods such as setting up searches, call and recall systems to contact patients, and targeted campaigns for specific vaccinations (eg flu).

"We run recalls [for adult migrant catch-up] constantly throughout the year. We will target separate cohorts of patients, just so we can make sure we're recalling everybody". Admin 5

For peer review only - http://bmjopen.bmj.com/site/about/guidelines.xhtml

Opportunistic usually meant identifying a patient needed a vaccination when they were attending the practice for another reasons. The vaccine could be given immediately, or the patient booked into an appointment at a later date.

"…if I notice and if I remember or have time to mention it, then I encourage people to… [but] they're usually coming with quite a few issues, and we're using an interpreter… there's a lot to cover…[hence no time to cover vaccination]. GP 18

There are also diverse approaches to vaccine delivery between practices, with different staff involved in different aspects of the vaccine programme. However, many programmes are nurse-led, with the practice nurse having main responsibility.

'[It's a] mixture of me, one of the partners, and then the reception staff are the ones who actually call the patients and arrange for them to come in" Admin 13

"If they're struggling to get somebody to agree [to take a specific vaccine]… we get the named GP.. to take responsibility for having that conversation and trying to talk them round" Admin 9

"…our vaccines are really well-run at the practice by one of the nurses in particular. She runs the whole immunisation program, the childrens, the flu, the catch up, everything. So, I would imagine that there's probably a lot going on that I'm not aware of. I suspect and she always goes on updates and is very much aware of new guidance to things so I'm sure that she's probably doing a lot of stuff behind the scenes that I'm not aware of." GP 3

Travel Vaccination and Occupational vaccines

Provision of catch-up vaccines and additional vaccines to adult migrants was also mentioned in the context of travel and occupational requirements. Delivery of travel vaccinations was highlighted by a variety of participants for migrants visiting their home countries and travelling to Haj.

For peer review only - http://bmjopen.bmj.com/site/about/guidelines.xhtml

"I think people are very good at knowing they need vaccinations, especially people who have been settled in England for quite a long time and are maybe making an infrequent return visit home to may visit relatives or family elders or to go for a celebration" Admin 6

"They will go for the bare minimum of what is offered, or what they need to have as certificated. If they're doing the pilgrim to the Haj, then they have to get the meningitis. If they… need yellow fever, they'll get the yellow fever…or they just don't have anything." PN 2

Different nationalities were reported as having varying levels of engagement with travel vaccine uptake. One PN reported Bangladeshi families travelling more being 'more engaged' than Middle Eastern people. Another reported Europeans as '*more engaged with travel clinics than...people… from Pakistan, India, Bangladesh and African countries". [PN2]* African patients were described as having a poorer uptake of travel clinics than Europeans "*people returning to DRC or Tanzania…their uptake is poorer than younger European people" PN2.*

Participants noted that travel clinics can also be an opportunity for opportunistic adult catch-up: *"The nurses who do the travel clinics are certainly very switched on to catch-up vaccines and will make sure everybody's up to date with DTP and MenACWY, even if they're not going to a country for which you need ACWY." GP 17*

Travel vaccines were often given privately due to recommendations these should be done outside of the core contract, and this was primarily the case for adults but not children.

"We do … Hepatitis A and then typhoid as part of the core contract. Anything else we direct patients to a private travel clinic" GP 24

However, there was variability in provision, with one GP stating: *"We don't charge for anything, including malaria pills" (GP 17). This would impact the "migrant population who are going backwards and forwards to their home countries [and] constitute quite a large percentage of patients that we see for travel clinics" (GP 17)*

For peer review only - http://bmjopen.bmj.com/site/about/guidelines.xhtml

Occupational vaccines were mentioned as sometimes being provided 'outside the schedule' for healthcare staff, such as nurses.

" [Hepatitis B] vaccination is not within the schedule, so it has to be treated like a private prescription…some of them are nurses …[and they] usually come from the South Indian population. Carers and nurses" GP 4

"We shouldn't be seeing people wanting occupational health-related vaccination, but we do often get people asking for that" PN 1

Strengthening vaccine delivery in UK primary care

Primary care staff raised a range of potential solutions and action points to increasing vaccine uptake, especially in adult migrants, including addressing personal, societal, and physical barriers to vaccination systems through UK primary care alongside financial incentives to primary care to deliver adult catch-up vaccination. Key barriers and respective solutions identified by participants have been summarised in Table 4.

Table 4: Barriers and solutions identified

Barrier	Potential solution	Key messages	Quotes	Profes sional
**Awareness of vaccination programmes for adults**	Community engagement, capacity development, investment and partnership-building to raise awareness	Engage with community leaders, faith groups to help GPs and public health systems to improve uptake for vaccines in migrants; provide opportunities for information sharing, outreach, engagement, communication	*'I …hope that the CCG have thought about this and have gone to local communities, through the mosque or through other social avenues to trying get [vaccine] uptake'*	GP 24
**Fear of authorities**	Community engagement to tackle mistrust; increasing trustworthiness	Education and raising awareness within communities to overcome fear and enable health-seeking	*'We have suggested … that they engage with the churches, that they obviously engage with information and advice, but it's a hard nut to*	GP 24

For peer review only - http://bmjopen.bmj.com/site/about/guidelines.xhtml

Barrier	Potential solution	Key messages	Quotes	Professional
	of health and other institutions	of preventative healthcare; (re)building trust through community engagement and investment	*crack if somebody's life is built around not trusting the specific institution.'*	
**Misinformation about vaccines**	Use trusted professionals or other trusted messengers - and ensure they are properly resourced, recognised and compensated(17)	One GP thought that consulting with someone who was felt to be an 'expert' in vaccinations would have better outcomes.	*'…If [the vaccine advice is from] from a GP…[or] from a consultant… then that tends to have a bit more weight to it… I think it depends on the level of education and understanding…'*	GP 25
	System approach - Building capacity to recognize and respond to misinformation; developing resources to increase health literacy;	Public Health messaging and a national approach	*I think it's got to be a national approach…We got the Public Health Department…'*	GP 22
	Patient education ; develop tailored messages	Patient education and sharing as much information as possible regarding vaccines, from all health professionals involved	*'People just need as much information as possible [about the vaccine], and I think information in particular on side effects etc"*	HCA 1
**Lack of training for staff around migrant health**	Staff education and training (both clinical and non-clinical staff)	Improving staff understanding of potential issues and communication skills	*It's just a bit of understanding… some patients may come across as difficult… [but with] extra training with staff…[understanding can improve]*	HCA 2

For peer review only - http://bmjopen.bmj.com/site/about/guidelines.xhtml

Barrier	Potential solution	Key messages	Quotes	Professional
**Financial pressures**	Financial payments and incentives	Including adult migrant vaccination targets as a financial incentive to ensure migrant adult catch-up programmes are carried out	*"…Unless they actually make [adult catch-up vaccination] something that they want GP surgeries to do, like proactively educate them and give them some renumeration to do it.. work is money and we haven't got enough practice nurses as it is…So it can't just be expected to be an add on'*	GP 18
**Lack of time**	Longer appointments	Longer appointments, especially if interpreter is needed	*We make the appointments longer*	PN 7
**Language barrier**	Interpreters; linguistically and culturally tailored information	Use interpreters for vaccine programmes, including written communication	*We sent out a lot of text messages [about vaccination]. That would be good if we could do those in different languages…*	PN 15
**Different vaccine schedules and lack of history**	Migrant specific health check	A health check for adult migrants, to gather information about vaccine history	*'[A] template which is specific for patients from different countries, which means that you're not trawling through evidence'*	GP 20
**Pressures on health system**	Ensure primary care deliver these vaccination programmes	Make migrant adult catch-up vaccination mandatory for primary care to provide	*'If they were part of QOF, they're made mandatory… that would definitely make [practices] do it'*	Pharmacist

Discussion

Key findings

WHO's new Immunization Agenda (IA2030)(9) has called for greater focus to be placed on delivering vaccination across the life-course, targeting under-immunised groups for catch-up vaccination at any age, with primary care services therefore having a key role to play in the UK

For peer review

context. In our study, however, participants highlighted direct and indirect barriers to delivering catch-up vaccines in adult migrants who may have missed vaccines as children, missed boosters, and not be aligned with the UK's vaccine schedule. Barriers were noted at a personal and service-delivery level, with themes including: lack of training and knowledge of guidance around catch-up vaccination among staff; unclear or incomplete vaccine records; and lack of incentivization (including financial reimbursement), prioritisation, and dedicated time and care pathways. Adult migrants were therefore reported as being excluded from many vaccination initiatives, most of which focus exclusively on children. In addition, PCPs reported that migrant patients express a range of views around vaccines to them, from positivity to uncertainty, to refusal. Some migrants including Somali, Eastern-Europeans and Bangladeshi groups were often reported as being hesitant to get vaccinated, with specific concerns reported for specific vaccines, including MMR but with more positive responses to travel vaccinations. Greater consideration needs to be placed on potential delivery points for catch-up vaccination in adult migrants – for example, local places of worship and other trusted or familiar sites – alongside offering financial incentives or inclusion of adult migrant vaccination targets in QOF. Improving uptake of catch-up vaccination in this group will require new care pathways and training of front-line staff, alongside working directly with local community groups to communicate the benefits of vaccination at all ages. In addition, greater collaboration across systems and community groups and culturally competent campaigns are warranted. At a time when COVID-19 vaccination programmes are being rolled-out across the world, this study adds important understanding regarding the specific vaccination needs and concerns of migrants, and the challenges faced by the staff delivering vaccination programmes to migrant populations and older cohorts.

Strengths and limitations

A key strength of the study is the number and variety of primary care staff included from across England in diverse settings. Interviewees were a self-selecting group, which may have affected the profile of those responding – a common consideration in qualitative research. However, a range of practices were involved, including those that do not see many migrants, and this diversity and the scale of the study is likely to have added to the breadth of experience and solutions reflected in our findings, as well as enhancing the validity. We noted that often participants made broad generalisations about specific nationality groups, which needs to be considered with commitment to equality, diversity and inclusion when assessing findings. The structure and experience of

For peer review only

primary care across Europe and between the devolved nations of the UK may differ so the recruitment only within England may limit the generalisability of the findings, however other European and international studies (7, 18, 19) have come to the similar conclusions in terms of healthcare provider, system, and patient-related barriers to catch-up vaccination in relation to adult migrants, so we feel that this would be unlikely.

Next steps for strengthening catch-up vaccination in older cohorts

We found a range of direct and indirect barriers to delivering catch-up vaccines in adult migrants who may have missed vaccines as children, missed boosters, and not be aligned with the UK's vaccine schedule, from both a personal and service-delivery level. Our findings concur with those of similar study in Norway (18) which found no consistent or structured approach to vaccinating adult migrants in Norway, including no guidelines from governing bodies on how to organise vaccination to adult migrants. Reasons why adult vaccination is not prioritised included tuberculosis screening and treatment taking precedence, and a common assumption among healthcare providers that vaccinations are dealt with in childhood(18). A questionnaire survey of experts across Europe(7), and policy analysis(20), found that policies and practice differ across European countries with respect to adult vaccination and the inclusion of migrants in vaccine systems on arrival. Only 13 of 32 countries in the EU/EEA had policies in place to offer MMR vaccines to adult migrants, with 10 countries reporting that they would charge fees(7). Variations in vaccine policies targeting adult migrants were reported in another European survey (21). In addition, it is well known that some migrants face a range of barriers to health systems more broadly. This suggests that more inclusive policies are required placing an emphasis on new approaches to ensure older migrants are included, and that such policies are well implemented in practice.

Implementation will be key, and our study raised numerous points that merit greater consideration. Service delivery barriers have previously been described in other areas of migrant health, including screening for infection, with GPs citing concerns about lack of awareness around the health needs of migrants and insufficient time and resources (22, 23). It has previously been noted that negative biases from healthcare staff towards migrant patients or pre-conceptions about vaccine hesitancy in specific ethnic groups may have an impact on patient trust (24, 25), which is known to be a major factor in vaccine uptake (26). Education and training of front-line providers will be a critical component given the critical role that the PCP-patient relationship has for building trust in

For peer review only - http://bmjopen.bmj.com/site/about/guidelines.xhtml

vaccination. This must involve raising awareness of the diverse experiences of migrants and how to approach potential vaccination concerns with sensitivity, as well ensuring an understanding around the potential the low vaccine coverage in their countries of origin as children, different dosing schedules, and particularly low coverage for newer vaccines. For HPV, for example, global coverage for the final dose was only 13% in 2021 (27) – suggesting many migrants aged under 25 years would be eligible for HPV vaccination as part of the UK's more advanced programme. However, likely a key factor will be financial incentivisation to encourage practices to target potentially under-immunised adults for catch-up vaccines, which was a recurrent theme among those interviewed. Catch-up vaccination could be considered at various entry points, for example the New Patient Health Check or the NHS Health Check. Since April 2020, MMR now comes with an item of service payment, including for catch-up vaccination in patients who missed out on scheduled vaccines, which should encourage practices to offer appropriate vaccinations to patients regardless of age.

Tackling hesitancy and educating migrant and broader ethnic minority communities about the benefits of vaccination across the life-course will also be a critical component(23, 28), with COVID-19 presenting numerous innovations in service delivery in this area that merit further consideration to routine vaccination going forward including outreach, policy shifts to facilitate registration of migrants with primary care providers, and anonymous vaccination in trusted locations (23, 29). We found that certain nationality groups (Somali, Eastern-Europeans and Bangladeshi) may be more hesitant to receive vaccines than others, or reluctant to receive certain vaccines, aligning with a recent systematic review that found nationality/country of origin to be a key determinant of vaccine uptake for routine vaccines and COVID-19 vaccines in European datasets (8). In this study, acceptance barriers were mostly reported in Eastern European and Muslim migrants for HPV, measles, and influenza vaccines, with 23 significant determinants of under-vaccination in migrants found ($p<0.05$), including African origin, recent migration, and being a refugee/asylum seeker (8). A systematic review of interventions to improve vaccination uptake in newly-arrived migrants to the EU/EEA (30) highlighted the potential solutions of social mobilization and outreach programmes, planned vaccinations, and educational campaigns. Our data points to a recommendation for policy makers to include adult migrants specially in catch-up vaccination programmes on arrival, and to ensure policy around the delivery of catch-up vaccination across the life-course is implemented in practice.

For peer review only

Contributors

JC and SH conceived the idea and developed the initial proposal. JC wrote the ethics applications, led the recruitment and data collection, and contributed to data analysis and manuscript revision. AM led the data analysis and contributed to the manuscript draft, revision, and concepts. FK contributed to data collection, data analysis, manuscript draft and revision. AD and AC contributed to the data collection and manuscript revision. AM, JC, FK, and SH wrote a first draft of the paper, with input from ACF, AD, LPG, FM, YC, AM.

Competing interests

Nothing to declare.

Funding

This study was funded by the National Institute for Health Research (NIHR) and the Academy of Medical Sciences. Felicity Knights is supported by a Health Education England/ NIHR Academic Clinical Fellowship. Jessica Carter is funded by an NIHR in-practice clinical fellowship (NIHR300290). The views expressed are those of the author(s) and not necessarily those of the NHS, the NIHR, or the Department of Health and Social Care. Anna Deal and Sally E Hayward are supported by Medical Research Council PhD studentships (reference number: MR/ N013638/1). Sally Hargreaves is funded by an NIHR Advanced Fellowship (reference number: NIHR300072), the Academy of Medical Sciences (reference number: SBF005\1111), the Novo Nordisk Foundation/La Caixa Foundation (Mobility–Global Medicine and Health Research grant), and the WHO. AC is funded by the Academy of Medical Sciences (SBF005\1111) and the NIHR (NIHR300072).

Data sharing statement

Abridged versions (to maintain full anonymity) of the datasets used and analysed during the study are available from the corresponding author on reasonable request.

Ethics statement

Ethical approval was granted by St George's, University of London Research Ethics Committee (reference numbers: REC 2020.0058 and 2020.00630) and the Health Research Authority (reference number: REC 20/HRA/1674).

For peer review only - http://bmjopen.bmj.com/site/about/guidelines.xhtml

References

1. Deal A, Halliday R, Crawshaw AF, Hayward SE, Burnard A, Rustage K, et al. Migration and outbreaks of vaccine-preventable disease in Europe: a systematic review. The Lancet Infectious Diseases. 2021.
2. Mipatrini D, Stefanelli P, Severoni S, Rezza G. Vaccinations in migrants and refugees: a challenge for European health systems. A systematic review of current scientific evidence. Pathogens and Global Health. 2017;111(2):59-68.
3. Dutta, T., Meyerson, B., & Agley, J. (2018). African cervical cancer prevention and control plans: A scoping review. Journal of cancer policy, 16, 73-81.
4. Jablonka A, Happle C, Grote U, Schleenvoigt BT, Hampel A, Dopfer C, et al. Measles, mumps, rubella, and varicella seroprevalence in refugees in Germany in 2015. Infection. 2016;44(6):781-7.
5. Norman FF, Comeche B, Martínez-Lacalzada M, Pérez-Molina J-A, Gullón B, Monge-Maillo B, et al. Seroprevalence of vaccine-preventable and non-vaccine-preventable infections in migrants in Spain. Journal of Travel Medicine. 2021;28(4).
6. Charania NA, Gaze N, Kung JY, Brooks S. Vaccine-preventable diseases and immunisation coverage among migrants and non-migrants worldwide: A scoping review of published literature, 2006 to 2016. Vaccine. 2019;37(20):2661-9.
7. Hargreaves S, Nellums LB, Ravensbergen SJ, Friedland JS, Stienstra Y, On Behalf Of The ESGITM Working Group On Vaccination In Migrants. Divergent approaches in the vaccination of recently arrived migrants to Europe: a survey of national experts from 32 countries, 2017. Eurosurveillance. 2018;23(41).
8. Crawshaw AF, et al. Defining the determinants of vaccine uptake and under-vaccination in migrant populations in Europe to improve routine and COVID-19 vaccine uptake: a systematic review. 2022 (Accepted, Lancet Infectious Diseases). https://www.medrxiv.org/content/10.1101/2021.11.08.21266058v1.
9. World Health Organization. Immunization Agenda 2030 2020 [updated 1 April 2020. Available from: https://www.who.int/teams/immunization-vaccines-and-biologicals/strategies/ia2030.
10. WHO. Leave no one behind. Guidance for planning and implementing catch-up vaccination. Geneva: WHO, 2021. .
11. World Health Organization (WHO). Immunization Agenda 2030: A Global Strategy to Leave No One Behind. 2020.
12. Noori T, Hargreaves S, Greenaway C, van der Werf M, Driedger M, Morton RL, et al. Strengthening screening for infectious diseases and vaccination among migrants in Europe: What is needed to close the implementation gaps? Travel Med Infect Dis. 2021;39:101715.
13. European Centre for Disease Prevention and Control. Public health guidance on screening and vaccination of migrants in the EU/EEA. Available from: https://www.ecdc.europa.eu/en/publications-data/public-health-guidance-screening-and-vaccination-infectious-diseases-newly. ECDC. Sweden: Stockholm; 2018.
14. Public Health England. Vaccination of individuals with uncertain or incomplete immunisation status. Updated 26th August 2021. Available from: https://www.gov.uk/government/publications/vaccination-of-individuals-with-uncertain-or-

For peer review only

incomplete-immunisation-status/vaccination-of-individuals-with-uncertain-or-incomplete-immunisation-status [Accessed 21st September 2021].
15. Morse JM (1995) The Significance of Saturation. Qual Health Res 5(2): 147-149 Available from: [https://journals.sagepub.com/doi/10.1177/104973239500500201]

16. Braun V, Clarke V. Thematic analysis. In: Cooper H, Camic PM, Long DL, editors. APA handbook of research methods in psychology, 2: research designs: quantitative, qualitative, neuropsychological, and biological. Washington, DC: American Psychological Association; 2012.
17. AuYOung M, et al. Addressing racial/ethnic inequities in vaccine hesitancy and uptake: lessons learned from the California alliance against COVID-19. J Behavioural Med 2022; 22nd Jan https://link.springer.com/article/10.1007/s10865-022-00284-8.
18. Socha A, Klein J. What are the challenges in the vaccination of migrants in Norway from healthcare provider perspectives? A qualitative, phenomenological study BMJ Open 2020;10:e040974. doi: 10.1136/bmjopen-2020-040974

19. Moussaoui S, Aurousseau AM, Nappez S, Cornaglia J, Delobre G, Blanchi S, et al. Immunization Catch-Up for Newly Arrived Migrants in France: A Cross-Sectional Study among French General Practitioners. 2021;9(6):21.
20. Ravensbergen SJ, et al. National approaches to the vaccination of recently arrived migrants in Europe: a comparative policy analysis across 32 European countries. Trav Med and Infect Dis 2019; 33-38. .
21. Giambi C, Del Manso M, Marchetti G, Olsson K, Adel Ali K, Declich S, et al. Immunisation of migrants in EU/EEA countries: Policies and practices. Vaccine. 2019;37(36):5439-51.
22. Evlampidou I, et al. Low hepatitis B testing among migrants: a cross-sectional study in a UK city. Br J Gen Pract 2016; 66)647): e382-e391. .
23. Crawshaw AF, Deal A, Rustage K, Forster AS, Campos-Matos I, Vandrevala T, et al. What must be done to tackle vaccine hesitancy and barriers to COVID-19 vaccination in migrants? Journal of Travel Medicine. 2021;28(4):01.
24. Jama A, et al. Perspectives on the measles, mumps and rubella vaccination among Somali Mothers in Stockholm. Int J Environmental Research and Public Health 2018; 15: 11.https://www.mdpi.com/1660-4601/15/11/2428.
25. Kasstan B. Vaccines and vitriol: an anthropological commentary on vaccine hesitancy, decision-making and interventionism among religious minorities. Anthrop & Med 2020; 13 Nov: 411-419
https://www.tandfonline.com/doi/full/10.1080/13648470.2020.1825618?casa_token=DiweSjBRwMQAAAAA%3Aw1JFAzqfbT8KsQTnz72bbpGy7pcmgRjehDA8OeGAMmOD1esZFPHL0IWhxq2M-p0vU5Z4F3-cFEaZTQ.
26. Larson HJ, et al. Understanding vaccine hesitancy around vaccines and vaccination from a global perspective: a systematic review of published literature, 2007-2012. Vaccine; 32: 19: 2150-2159.
27. WHO. Immunization coverage. July 15, 2021. Available from: https://www.who.int/news-room/fact-sheets/detail/immunization-coverage.
28. Tankwanchi AS, Bowman B, Garrison M, Larson H, Wiysonge CS. Vaccine hesitancy in migrant communities: a rapid review of latest evidence. Curr Opin Immunol. 2021;71:62-8.
29. Doctors of the World UK. Vaccine Confidence Toolkit. England: London.2021. [Available from: https://www.doctorsoftheworld.org.uk/what-we-stand-for/supporting-medics/vaccine-confidence-toolkit/

30. Hui, C., Dunn, J., Morton, R., Staub, L. P., Tran, A., Hargreaves, S., Greenaway, C., Biggs, B. A., Christensen, R., & Pottie, K. (2018). Interventions to Improve Vaccination Uptake and Cost Effectiveness of Vaccination Strategies in Newly Arrived Migrants in the EU/EEA: A Systematic Review. International journal of environmental research and public health, 15(10), 2065. https://doi.org/10.3390/ijerph15102065.

For peer review only

Standards for Reporting Qualitative Research (SRQR)*

http://www.equator-network.org/reporting-guidelines/srqr/

Title and abstract

	Page/line no(s).
**Title** - Concise description of the nature and topic of the study Identifying the study as qualitative or indicating the approach (e.g., ethnography, grounded theory) or data collection methods (e.g., interview, focus group) is recommended	1
**Abstract** - Summary of key elements of the study using the abstract format of the intended publication; typically includes background, purpose, methods, results, and conclusions	2

Introduction

**Problem formulation** - Description and significance of the problem/phenomenon studied; review of relevant theory and empirical work; problem statement	6-7
**Purpose or research question** - Purpose of the study and specific objectives or questions	6-7

Methods

**Qualitative approach and research paradigm** - Qualitative approach (e.g., ethnography, grounded theory, case study, phenomenology, narrative research) and guiding theory if appropriate; identifying the research paradigm (e.g., postpositivist, constructivist/ interpretivist) is also recommended; rationale**	8-10
**Researcher characteristics and reflexivity** - Researchers' characteristics that may influence the research, including personal attributes, qualifications/experience, relationship with participants, assumptions, and/or presuppositions; potential or actual interaction between researchers' characteristics and the research questions, approach, methods, results, and/or transferability	10
**Context** - Setting/site and salient contextual factors; rationale**	9
**Sampling strategy** - How and why research participants, documents, or events were selected; criteria for deciding when no further sampling was necessary (e.g., sampling saturation); rationale**	10
**Ethical issues pertaining to human subjects** - Documentation of approval by an appropriate ethics review board and participant consent, or explanation for lack thereof; other confidentiality and data security issues	8
**Data collection methods** - Types of data collected; details of data collection procedures including (as appropriate) start and stop dates of data collection and analysis, iterative process, triangulation of sources/methods, and modification of procedures in response to evolving study findings; rationale**	10

For peer review only - http://bmjopen.bmj.com/site/about/guidelines.xhtml

For peer review only

**Data collection instruments and technologies** - Description of instruments (e.g., interview guides, questionnaires) and devices (e.g., audio recorders) used for data collection; if/how the instrument(s) changed over the course of the study	10
**Units of study** - Number and relevant characteristics of participants, documents, or events included in the study; level of participation (could be reported in results)	11
**Data processing** - Methods for processing data prior to and during analysis, including transcription, data entry, data management and security, verification of data integrity, data coding, and anonymization/de-identification of excerpts	10
**Data analysis** - Process by which inferences, themes, etc., were identified and developed, including the researchers involved in data analysis; usually references a specific paradigm or approach; rationale**	10
**Techniques to enhance trustworthiness** - Techniques to enhance trustworthiness and credibility of data analysis (e.g., member checking, audit trail, triangulation); rationale**	10

Results/findings

**Synthesis and interpretation** - Main findings (e.g., interpretations, inferences, and themes); might include development of a theory or model, or integration with prior research or theory	11-22
**Links to empirical data** - Evidence (e.g., quotes, field notes, text excerpts, photographs) to substantiate analytic findings	11-22

Discussion

**Integration with prior work, implications, transferability, and contribution(s) to the field** - Short summary of main findings; explanation of how findings and conclusions connect to, support, elaborate on, or challenge conclusions of earlier scholarship; discussion of scope of application/generalizability; identification of unique contribution(s) to scholarship in a discipline or field	23-25
**Limitations** - Trustworthiness and limitations of findings	23-24

Other

**Conflicts of interest** - Potential sources of influence or perceived influence on study conduct and conclusions; how these were managed	26
**Funding** - Sources of funding and other support; role of funders in data collection, interpretation, and reporting	26

*The authors created the SRQR by searching the literature to identify guidelines, reporting standards, and critical appraisal criteria for qualitative research; reviewing the reference lists of retrieved sources; and contacting experts to gain feedback. The SRQR aims to improve the transparency of all aspects of qualitative research by providing clear standards for reporting qualitative research.

For peer review only - http://bmjopen.bmj.com/site/about/guidelines.xhtml

**The rationale should briefly discuss the justification for choosing that theory, approach, method, or technique rather than other options available, the assumptions and limitations implicit in those choices, and how those choices influence study conclusions and transferability. As appropriate, the rationale for several items might be discussed together.

Reference:

O'Brien BC, Harris IB, Beckman TJ, Reed DA, Cook DA. **Standards for reporting qualitative research: a synthesis of recommendations.** *Academic Medicine*, Vol. 89, No. 9 / Sept 2014 DOI: 10.1097/ACM.0000000000000388

For peer review only
